# Paclitaxel Response Can Be Predicted With Interpretable Multi-Variate Classifiers Exploiting DNA-Methylation and miRNA Data

**DOI:** 10.3389/fgene.2019.01041

**Published:** 2019-10-25

**Authors:** Alexandra Bomane, Anthony Gonçalves, Pedro J. Ballester

**Affiliations:** Cancer Research Center of Marseille, CRCM, INSERM, Institut Paoli-Calmettes, Aix-Marseille Univ, CNRS, Paris, France

**Keywords:** biomarker discovery, machine learning, artificial intelligence, precision oncology, tumor profiling

## Abstract

To address the problem of resistance to paclitaxel treatment, we have investigated to which extent is possible to predict Breast Cancer (BC) patient response to this drug. We carried out a large-scale tumor-based prediction analysis using data from the US National Cancer Institute’s Genomic Data Commons. These data sets comprise the responses of BC patients to paclitaxel along with six molecular profiles of their tumors. We assessed 10 Machine Learning (ML) algorithms on each of these profiles and evaluated the resulting 60 classifiers on the same BC patients. DNA methylation and miRNA profiles were the most informative overall. In combination with these two profiles, ML algorithms selecting the smallest subset of molecular features generated the most predictive classifiers: a complexity-optimized XGBoost classifier based on CpG island methylation extracted a subset of molecular factors relevant to predict paclitaxel response (AUC = 0.74). A CpG site methylation-based Decision Tree (DT) combining only 2 of the 22,941 considered CpG sites (AUC = 0.89) and a miRNA expression-based DT employing just 4 of the 337 analyzed mature miRNAs (AUC = 0.72) reveal the molecular types associated to paclitaxel-sensitive and resistant BC tumors. A literature review shows that features selected by these three classifiers have been individually linked to the cytotoxic-drug sensitivities and prognosis of BC patients. Our work leads to several molecular signatures, unearthed from methylome and miRNome, able to anticipate to some extent which BC tumors respond or not to paclitaxel. These results may provide insights to optimize paclitaxel-therapies in clinical practice.

## Introduction

Breast cancer (BC) is the most common type of cancer in women worldwide resulting in half a million deaths annually (Golubnitschaja et al., 2016). BC is a disease presenting substantial inter-tumor heterogeneity (Russnes et al., 2011). Cytotoxic drugs are used to eradicate tumor cells, to complement surgery or radiotherapy as well as to alleviate cancer symptoms. Paclitaxel is a BC-approved cytotoxic drug from the taxane family, which acts by interfering with the normal function of microtubules during cell division (Perez, 1998). As with other cancer drugs (Brown and Böger-Brown, 1999; Cardoso et al., 2002; Ribeiro et al., 2012; Housman et al., 2014), resistance to paclitaxel have been regularly observed in BC patients (Flint et al., 2009; Ajabnoor et al., 2012).

Precision oncology requires predictors to guide the optimization of drug therapies for patients (Peck, 2016; Schwartzberg et al., 2017). Indeed, it is now well-established that gene polymorphisms and other genomic alterations play important roles in the observed heterogeneous response to drugs (Wang et al., 2011;Harper and Topol, 2012; Kadra et al., 2012). This has led to the identification of clinical biomarkers of drug response from molecular profiles of the patients’ tumors (Huang et al., 2014). These predictive biomarkers now guide patient-specific treatment selection during clinical trials and are also used in clinical practice (Mandrekar and Sargent, 2009; Biankin et al., 2015). Most commonly, single-gene markers are used to discriminate between therapy responders and non-responders (Prahallad et al., 2012; Rodríguez-Antona and Taron, 2015), typically consisting of an actionable mutation (e.g. single-nucleotide variant) of a specific gene in the tumor sample.

Single-gene markers that are able to predict the efficacy of cytotoxic drugs are rare (Felip and Martinez, 2012), especially for taxanes (Murray et al., 2012; Bartlett et al., 2015; Norimura et al., 2018). For instance, Marsh et al. (2007) have proposed that the point mutation CYP1B1*3 could be an important factor that helps to differentiate between sensitive and resistant BC patients to paclitaxel. However, Gehrmann et al. (2008) have raised doubts about the association between this alteration and paclitaxel-treated patient prognosis, concluding that CYP1B1 alone is not sufficient to predict tumor response to paclitaxel, and that it could interact with still unknown factors involved in paclitaxel sensitivity. This is an example of inter-patient variability in drug response not being fully captured by the mutational status of single gene, as it has also been seen *in vitro* in a range of drugs (Naulaerts et al., 2017).

Machine Learning (ML) can be used to build *in silico* models able to predict tumor response to a given drug by combining multiple tumor features in an optimal manner (Libbrecht and Noble, 2015; Ali and Aittokallio, 2018). The scarcity of suitable clinical data to build such predictors has been a major roadblock, which has made predictors based on cancer cell line data thrive (Costello et al., 2014; Ding et al., 2016). Fortunately, response data from paclitaxel-treated BC patients along with comprehensive molecular profiles of their tumors are increasingly available. Such datasets represent an opportunity to improve our ability to anticipate which BC patients will respond to paclitaxel. We obtained them from the recently created Genomic Data Commons (GDC) of the US National Cancer Institute (NCI) (Jensen et al., 2017). The GDC provides a unified data repository enabling data sharing across cancer genomic studies in support of precision medicine. The GDC feeds from several cancer genome programs at the NCI Center for Cancer Genomics, notably The Cancer Genome Atlas (TCGA) (Weinstein et al., 2013), and offers a range of information-rich genomic, transcriptomic and epigenomic profiles, as well as clinical drug response data.

These datasets, however, pose the challenge of being high-dimensional. Each profile typically contains between hundreds and many thousands of features, but only tens of profiled tumors of the same cancer type and treated with the same drug. For example, a community challenge intended to predict drug response employed 53 BC cell lines (Costello et al., 2014), while thousands of features from DNA copy-number variation, transcript expression, mutations, DNA methylation, and protein abundance profiles were considered. In another study (Tripathi et al., 2016), predictive models of response to cytotoxic drugs were achieved using 60 pancancer cell lines and gene variants as features. A further example of predictive drug-sensitivity models is a study employing 60 diverse cell lines and protein abundances as features (Ma et al., 2006). Small sample sizes are not only typical of preclinical studies, but also of clinical studies addressing the same problem. For instance, gene expression signatures were identified and evaluated using 81 melanoma patients to predict their response to PD-1 checkpoint inhibitors (Ayers et al., 2017).

In this study, we will investigate whether it is possible to anticipate the response of BC patients to paclitaxel using GDC data. We also aim at discovering the molecular factors that, collectively, best discriminate between paclitaxel-resistant and paclitaxel-sensitive BC patients. High-dimensional data promotes model overfitting, which in turn results in poorer predictions. As predictive performance differences between ML algorithms are strongly problem-dependent (Tan and Gilbert, 2003; Fernández-Delgado et al., 2014), considering a range of algorithms to identify those that are most suitable for paclitaxel-treated BC patients is appealing. To this end, we apply 10 ML methods to build predictive models in combination with each available molecular profile. Some of the resulting multi-variate predictors are highly interpretable in that they can answer questions such as why this particular patient is non-responsive. This information should permit formulating hypothesis about the molecular mechanisms of BC patient resistance to paclitaxel.

## Material and Methods

### GDC Data

GDC molecular profiling and clinical data from the TCGA Breast Invasive Carcinoma or BRCA (https://portal.gdc.cancer.gov/projects/TCGA-BRCA) provide the basis for this study. Molecular profiles and clinical data come from release version 4.0, except for miRNA and miRNA isoform (isomiR) expressions coming from release version 8.0 (Release Notes- GDC Docs).

TCGA-BRCA project gathers data from 1,098 patients, resulting in almost 13,000 files (around 130 GB). These datasets were retrieved and downloaded using the GDC Application Programming Interface (API). **Table S1** reports information about files collected from the GDC that have been used to generate datasets.

### Processing Clinical Data for Modelling

Patient population included primary or secondary advanced breast cancer receiving single-agent paclitaxel. For some patients it was observed that different drugs have the same or very close treatment start and end time. These entries may form part of a drug combination. However, available drug response annotations do not allow to check this information. Therefore, possible effects due to drug combinations are ignored in this study when identifying paclitaxel-treated patients. Patients with missing paclitaxel response were not retained. To only consider baseline tumors’ molecular profiles, patient records were only retained if no treatment was administered before resection and the time of sample procurement is indicated. We assumed that a baseline molecular profile can explain drug responses observed in a given patient even if paclitaxel was administered at any time after sample resection (Geeleher et al., 2014). After these curation steps, 61 paclitaxel-treated BC patients with valid records remained (**Table S2** reports information about treatments and biospecimens). Annotated patient responses are divided into four categories based on the RECIST standard (Therasse et al., 2000): “Complete Response” (CR), “Partial Response” (PR), “Stable Disease” (SD), and “Clinical Progressive Disease” (CPD). We further classified clinical responses into two categories, namely “responder” (CR or PR) and “non-responder” (SD or CPD).

### Processing Molecular Profiles for Modelling

The GDC works on harmonization of raw genomic data developing specific workflows to provide consistent and up-to-date molecular profiles (GDC Reference Files| NCI Genomic Data Commons, Genomic Data Harmonization| NCI Genomic Data Commons). Available profiles comprise copy-number variation (CNV), DNA methylation, mRNA, miRNA and isomiR (Ameres and Zamore, 2013) expressions. In order to produce suitable inputs for ML algorithms and/or extract some specific information, we processed some of them. More details are available in the homonym section of Supplementary Methods. All the datasets produced from these molecular profiles are made of real-valued features.

### Predicting Drug Response Using ML Algorithms With Embedded Feature Selection

Most classifiers were built with ML algorithms capable of embedded feature selection to mitigate the impact of high-dimensional data on their generalization on unseen data. Implementations of Classification And Regression Tree (CART) (Breiman et al., 1984) and Random Forest (RF) (Breiman, 2001) were taken from the python library *Scikit-learn* version 0.19.1, while the ones of XGBoost (XGB) (Chen and Guestrin, 2016) version 0.6 and LightGBM (LGBM) (Ke et al., 2017) version 2.0.10 were respectively downloaded from https://github.com/dmlc/xgboost and https://github.com/Microsoft/LightGBM. We also applied a Deep Neural Network (DNN) algorithm (Bengio, 2009) implemented with the python library *Keras* version 2.2.4 using the TensorFlow backend. In addition to these nonlinear models, linear models were generated with Logistic Regression (LR) (Ranstam et al., 2016), which is also implemented in *Scikit-learn*. The visualization of Decision Trees (DTs) was done with the python package *dtreeviz* version 0.2.2. The homonym section in Supplementary Methods provides more details.

### Predicting Drug Response Using the Optimal Model Complexity (OMC)

OMC is a strategy to build ML models employing only the most relevant features (Nguyen et al., 2018). More details are available in the homonym section of **Supplementary Methods**.

### Measuring the Predictive Performance of a Classifier

This is a binary classification problem, as each patient belongs to one of two classes, responder and non-responder, with the responder considered as the positive class. As it is customary with problems with a small number of data instances (**Table S3**), we are using LOO (Leave-One-Out) CV (Cross-Validation) to evaluate the developed classifiers. Several types of LOOCV will be used: standard for “all-features models”, nested for “OMC models”, and permutated for “permutation models”. As with any other CV (Kohavi, 1995), each data instance (patient here) is exactly once in the test set. Thus, CV performance of a model is exclusively based on the prediction of test instances that were not used in any way to train or select the model (any feature selection is hence carried out on training folds only). Employing nested CV on algorithms requiring model selection (those employing OMC) provides an unbiased estimate of model performance, as it has been shown elsewhere (Cawley and Talbot, 2010; Varma and Simon, 2006).

Once known and predicted classes are compared for all held-out samples, true positives (TP), true negatives (TN), false positives (FP) and false negatives (FN) are counted among BC patients. These counts are used for calculating classification metrics that summarize the predictive performance reached by a classifier: precision, recall, F1-scores (Van Rijsbergen, 1979), and Matthews Correlation Coefficient (MCC) (Matthews, 1975; Boughorbel et al., 2017). More details about these metrics can be found in the homonym section of Supplementary Methods. Classification scores and contingency matrices obtained from all produced classifiers are stored in **Tables S4** and **S5**, respectively.

## Results

### Benchmarking of All-Features Models (RF, XGB, LGBM, DNN, LR) Reveals Some Informative Molecular Profiles, But the Resulting Classifiers Perform Marginally Better Than Random

**Figure 1:**
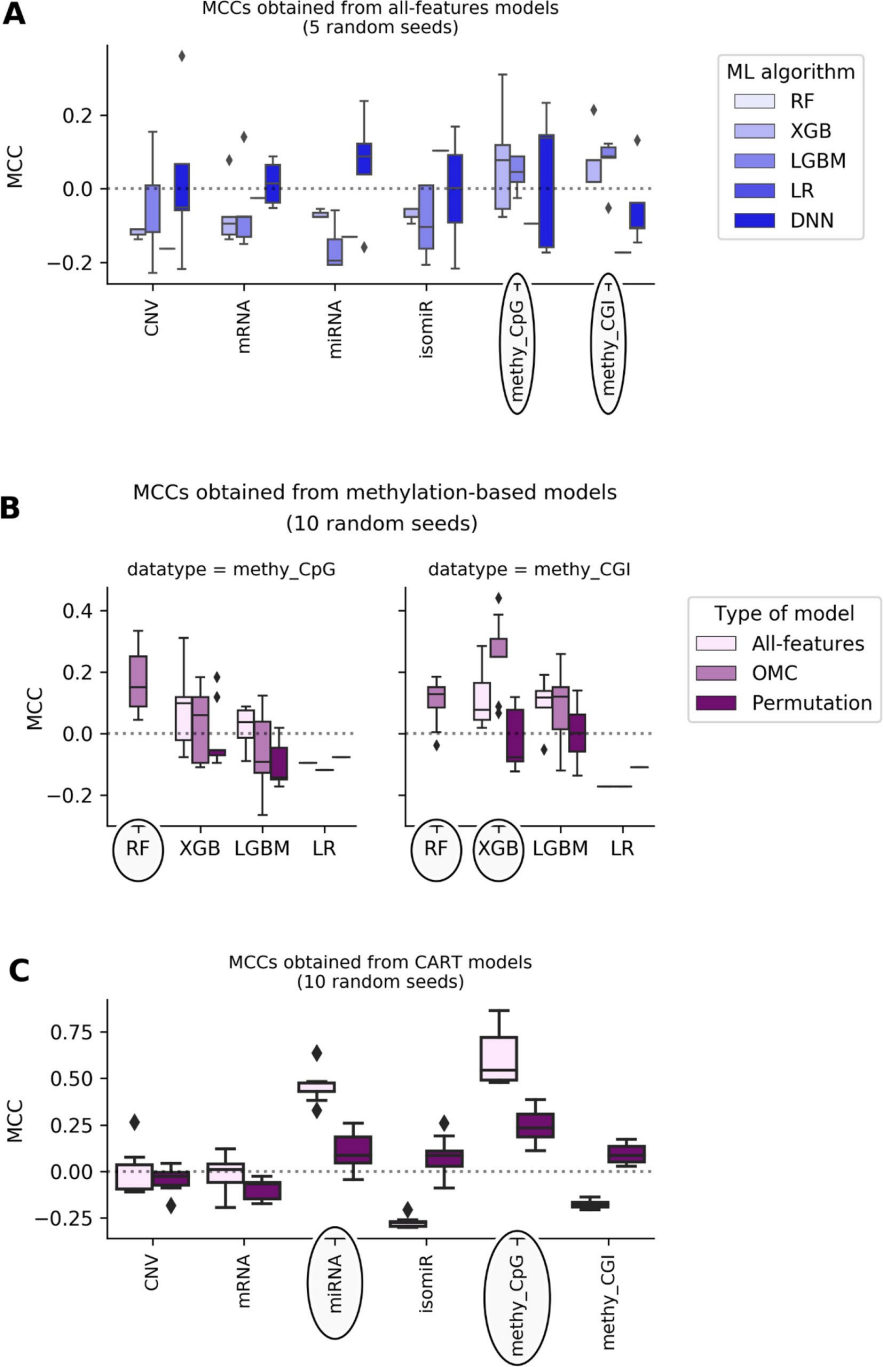
DNA methylation and miRNA expression lead to the most predictive ML models. Each MCC of a given model is calculated by LOOCV. The experiment is repeated several times, each time with a different random seed, giving rise to a boxplot of MCCs for each case. Permutation models were generated after shuffling class labels on the considered training set. As RF models give undefined MCCs, blanks are found in bins where boxplots are expected. Substantial variability is observed, showing that this problem is both profile- and classification method-dependent. The dashed line shows the expected MCC from random classification. **(A)** Predictive performance of all-features models. All-features models are those in principle employing all the features in the profile to generate the prediction. Models are built with ML algorithms using the default operating threshold (0.5) to calculate the predicted class label from the predicted class probability. Five random seeds were set for each ML algorithm; thus, MCC values come from five runs of standard LOOCV. *x*-axis shows the employed molecular profile, while *y*-axis displays the MCCs obtained by classifiers. From the lightest to the darkest blue, boxplots summarize the distributions of MCCs obtained by XGB,LGBM, LR, and DNN models, respectively. Ellipses indicate which profiles employed by models obtain better-than-random predictive performance: DNA methylation profiles are the most predictive. This also suggests that the other profiles are less informative for the prediction of BC tumor response to paclitaxel. **(B)** Predictive performance applying OMC to methylation-based models. 10 random seeds were used to investigate further the most predictive profiles. OMC models had their hyperparameters complexity and operating threshold tuned and thus required nested LOOCV. Horizontal axes show the employed ML algorithms to process CpG site (left) and CGI (right) methylation datasets, while vertical axes display MCCs achieved by classifiers. Light-pink, medium-pink, and indigo boxplots summarize the distributions of MCCs obtained by all-features, OMC and permutations models, respectively. Circles indicate ML algorithms releasing models with predictive performance improved using OMC. This shows that predictive accuracy depends on both the molecular profile and the ML algorithm. Here, the best models found are CpG site methylation-based RF-OMC, CGI methylation-based RF-OMC, and CGI methylation-based XGB-OMC. **(C)** Predictive performance of CART models. These models (light-pink boxes) were built considering all features in the profile with no hyperparameter tuning. Permutation models (indigo boxes) were trained after that shuffling class labels in the training set. Each MCC is calculated by standard LOOCV, a process repeated with 10 different random seeds. *x*-axis shows the molecular profiles (‘CNV’ is short for copy-number variation, ‘methy_CpG’ for CpG site methylation, and ‘methy_CGI’ for CGI methylation), while *y*-axis displays the LOOCV MCCs achieved by each classifier. The dashed line shows the expected MCC from random classification. Ellipses indicate molecular profiles processed by CART models obtaining the highest predictive performance. These results reveal that CpG sites methylation-based and miRNA expression-based CART models are the most predictive. Predictive accuracy is substantially higher than that provided by all-features or OMC models (in **A** and **B**), which demonstrates that the CART learning algorithm is more suitable for these problem instances.

**Figure 1** shows that most of the all-features ML classifiers perform worse than random classification reaching slightly negative median MCCs (from -0.19 to -0.05). Poor performance was also obtained when using linear models: LR models perform randomly at best (median MCCs range from -0.17 to 0.1 depending on the profile). Poor predictive performance is primarily caused by FPs (i.e. misclassification of non-responders). This problem was particularly noticeable in RF models, which misclassified every non-responsive patient regardless of the employed profile, leading to undefined MCCs. The latter shows that all-features RF handles class imbalance poorly on these particular problem instances.

DNA methylation-based XGB, LGBM, and DNN models achieve median MCCs slightly higher than 0.0, and they perform hardly better than permutation models. On the one hand, CpG site methylation-based XGB, LGBM, and DNN models obtain a median MCC of 0.08 (*p*-value from two-sided paired Student’s *t*-test obtained by class-permutation test = 1.05·10^-1^), 0.04 (*p*-value = 1.41·10^-1^), and 0.14 (*p*-value = 4.08·10^-1^), respectively. On the other hand, CpG island (CGI) methylation-based XGB and LGBM models achieve a median MCC of 0.08 (*p*-value = 2.33·10^-1^) and 0.09 (*p*-value = 8.04·10^-2^), respectively. Moreover, miRNA and mRNA expression-based DNN models had a median MCC of 0.088 (*p*-value = 4.84·10^-2^) and 0.015 (*p*-value = 4.76·10^-1^), respectively.

### Complexity-Optimized ML Models (RF-OMC, XGB-OMC, and LGBM-OMC) Provide Better Prediction and Extract Relevant Factors for Paclitaxel Response From CGI Methylation Data

Using OMC allows both to reduce considerably the number of features considered during model training and to adjust the operating threshold for assigning class labels to data instances. This leads to some OMC models that perform better than those considering all features from dataset (**Table S12** in **Supplementary Results**). This is especially the case for some methylation-based models that have been improved using OMC (**Figure 1**), unlike for models based on other profiles (**Figure S5**). The improvement of OMC over the all-features approach is ML algorithm-dependent.

CGI methylation-based OMC models have obtained improved predictive performance, using either RF or XGB. For instance, XGB-OMC models obtain a median MCC of 0.25, which is significantly better than both permutation and all-features models (*p*-values equal 9.30 ×·10^-4^ and 2.16 ×·10^-2^, respectively). In order to extract a robust subset of molecular factors potentially involved in paclitaxel response, the most informative features selected by these models were investigated (**Table S13** and **S14**). It results in 7 out of the 11,644 CGI coordinates encoded as CGI_ID.24217, CGI_ID.15915, CGI_ID.6919, CGI_ID.5276, CGI_ID.5459, CGI_ID.16043, and CGI_ID.11903. Moreover, we notice that 5 of them are common to the features used by the RF-OMC models. Consulting indices provided in **Tables S7** and **S8** (more details in **Supplementary Methods**), we found that these coordinates are related to the following 16 genes: CYP2D6, NDUFA6-AS1, RP4-669P10.19 (or C6orf108 pseudogene), MBTPS2, YY2, C2orf40 (or ECRG4), UXS1, IKZF1, APOBEC4, RGL1, ARPC5, NCF2, SMG7, C1orf177 (or LEXM), RP11-631M21.6 (or FAM166A pseudogene 7), and TUBB8 (**Table S14**).

### Transparent ML Models (CART) Capture CpG Methylation Sites and Mature MIRNAS Relevant for the Sensitivity to Paclitaxel and Show How They Are Combined to Explain Drug Response

Most of the available profiles led to poor classification of test set patients when modelled with CART (**Figure 1**). By contrast, CART classifiers based on miRNA expression and CpG site methylation data provided high to very high predictive performance in the context of this problem (in 10 LOOCV runs, median MCCs of 0.43 and 0.54 were obtained, respectively) and performed significantly better than random models (*p*-values from two-sided paired Student’s *t*-test obtained by class-permutation test equal 4.57·× 10^-6^ and 2.86·× 10^-4^, respectively; see **Figure 1** and **Table 1**). For each case, the best model is defined as that obtaining the highest MCC in 10 standard LOOCVs of the full dataset (i.e. all data instances and all available features). **Figure S6** shows that the performance of these models is robust to different sizes of both training set and test set.

**Table 1:** Best CART models.

Tumor profiling data	Number of considered features	Number of selected features	Median MCC(CART trained on original data)	Median MCC(CART trained on class-permutated data)	*p*-value(original vs permutated)
miRNA	337	4	0.43	0.09	4.57·10^-6^
methy_CpG	22,941	2	0.54	0.23	2.86·10^-4^

The predictive performance of CART models was presented in **Figure 1**. Here we summarize the characteristics of the two best models (i.e. those exploiting miRNA expression and CpG methylation profiles). A median MCC was calculated with the 10 MCCs coming from LOOCV experiments (each with a different random seed). This five additional LOOCV runs with respect to those presented in **Figure 3** were carried out to better characterize the performance of the best models found in our study. The small difference found in median MCC (0.52 in **Figure 3** versus 0.54 here) suggests that this performance metric is quite robust to the number of LOOCV runs for CART. The training sets were also class-permutated during cross-validation as explained in the Methods section and CART trained on the resulting data to provide a second set of 10 MCCs per profile. The p-value (two-sided paired Student’s t-test) of this class-permutated test shows how likely are the MCCs of the CART models to arise by chance. The first model was trained on miRNA expression: 4 out of 337 mature miRNAs were retained to build this model reaching a median MCC of 0.43 and performing significantly better than models based on permutated data (p-value = 4.57·10^-6^). The second model is obtained processing CpG site methylation (shorten as ‘methy_CpG’): 2 out of 22,941 CpG sites were retained to build this model achieving a median MCC of 0.54 and performing significantly better than permutation models (p-value = 2.86·10^-4^).

**Figure 2:**
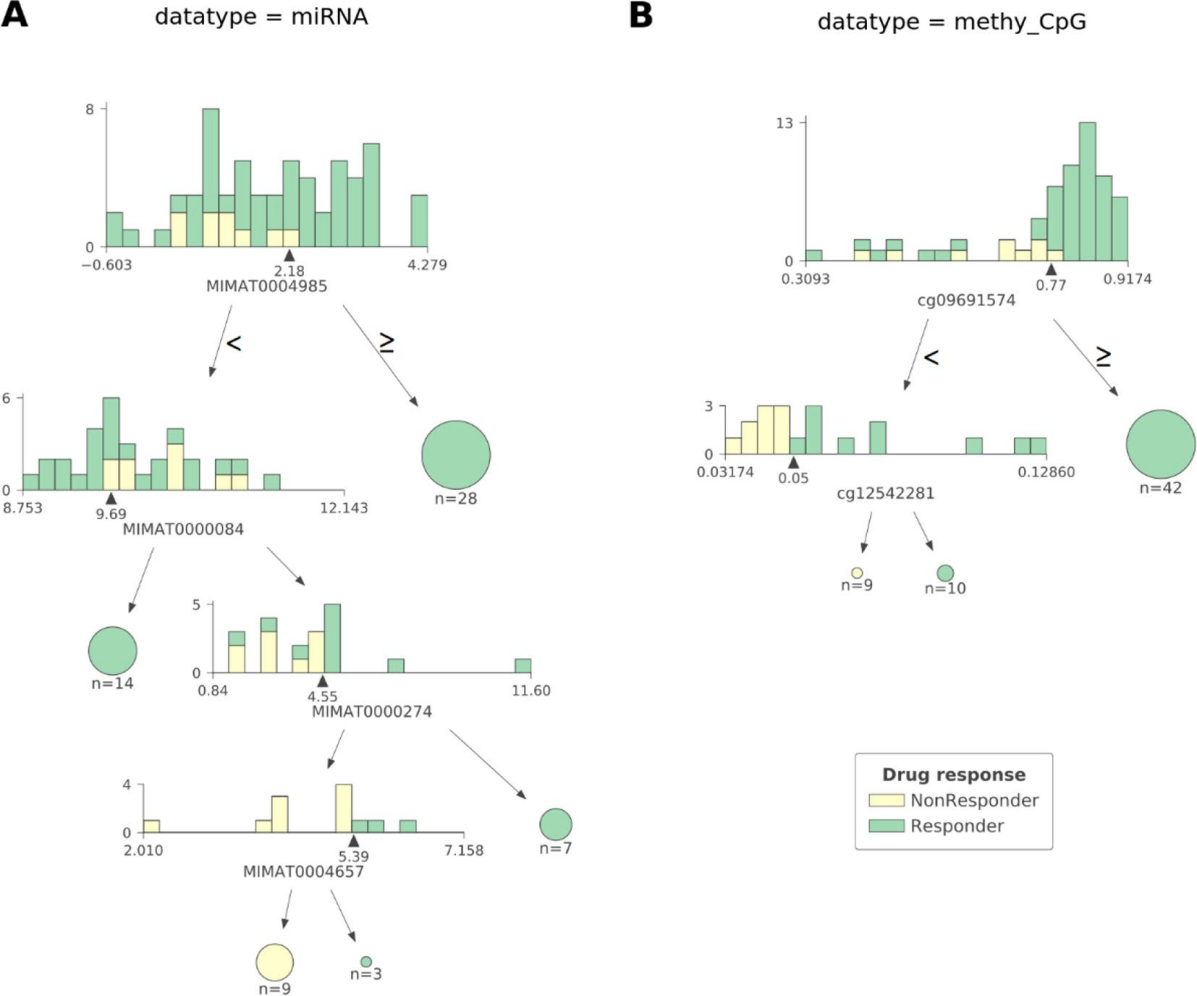
The most predictive CART models offer high interpretability of BC tumors response to paclitaxel. Visualization of the most predictive CART models seen in **Figure 1** exploiting **(A)** miRNA expression and **(B)** CpG site methylation data. Each DT node has a histogram showing the distribution of patients at that node against the selected feature (the proportion of responders vs non-responders in each feature bin is also shown). The triangle under the histogram marks the value of the best split for the selected feature, whose name can be found under the histogram as well. Each node has two leaves: to the left (patients with a feature value lower than that of the best split) and to the right (the rest of the patients). Terminal nodes (or leaves) are displayed as pie charts. The proportions of non-responders and responders are respectively colored yellow and green. The log2-transformed miRNA expression-based DT shown in **(A)** reveals four different molecular types of sensitive BC tumors and one molecular type associated to resistant BC tumors involving four mature miRNAs: MIMAT0004985, MIMAT0000084, MIMAT0000274, and MIMAT0004657 (also known as miR-942-5p, miR-27a-3p, miR-217, and miR-200c-5p, respectively). Thus, a tumor is classified as responsive to paclitaxel if: 1) the expression value of MIMAT0004985 is higher than 2.18, or 2) the expression value of MIMAT0004985 is lower than 2.18 and that of MIMAT0000084 is lower than 9.69, or 3) the expression value of MIMAT0004985 is lower than 2.18, and that of MIMAT0000084 is higher than 9.69 and that of MIMAT0000274 is higher than 4.55, or 4) the expression value of MIMAT0004985 is lower than 2.18, and that of MIMAT0000084 is higher than 9.69, and that of MIMAT0000274 is lower than 4.55, and that of MIMAT0004657 is higher than 5.39. Otherwise, the tumor is classified as non-responsive. The DT based on CpG site methylation (shortened as ‘methy_CpG’) shown in **(B)** unveils two different molecular types of sensitive BC tumors and one type of resistant BC tumors involving two CpG sites represented by probes cg09691574, which is related to the protein coding genes, MRGPRX4 and SAA2-SAA4; and to the lincRNA RP11-113D6.6, also called antisense, to MRGPRX4; and to cg12542281, which is related to the protein coding gene N4BP2L2. Thus, a tumor is predicted to be sensitive to paclitaxel if: 1) the beta value associated to the methylation of cg09691574 is higher than 0.77, or 2) the beta value associated to the methylation of cg09691574 is lower than 0.77, and that of cg12542281 is higher than 0.05. Otherwise, the tumor is predicted to be resistant. Both DTs were found to give pure leaves (i.e. all data instances that are in terminal nodes belong to the same class).

As observed in **Figure 2** CART models strongly reduce the number of features involved in the predictions. The miRNA expression-based model found that 4 out of 337 mature miRNAs were the most informative features (MIMAT0004985 or miR-942-5p, MIMAT0000084 or miR-27a-3p, MIMAT0000274 or miR-217, and MIMAT0004657 or miR-200c-5p), while the CpG-site methylation model identified 2 out of 22,941 CpG sites as the most informative features (cg09691574, which is related to the protein coding genes MRGPRX4 and SAA2-SAA4, and to the lincRNA RP11-113D6.6 also called antisense to MRGPRX4; and cg12542281, which is related to the protein coding gene N4BP2L2). The DTs represented in **Figure 2** show directly the interactions between independent features leading to the predictions. They also reveal the molecular types associated to paclitaxel-sensitive and paclitaxel-resistant BC tumors (the CpG site index is provided in **Table S7**).

Lastly, integrating different molecular profiles has sometimes been found to provide small predictive accuracy gains, e.g. see **Figure 4** in this study (Costello et al., 2014). Thus, since both miRNA and methy_CpG profiles led to the most predictive models, it makes sense to integrate these data sets and train CART models on the features of the resulting hybrid profile. Using the same 10 random seeds as the methy_CpG-based CART models (median LOOCV MCC of 0.54), the hybrid CART models obtained slightly worse accuracy (median LOOCV MCC of 0.52). The resulting CART tree is identical to that in **Figure 2**, suggesting that miRNA features were overshadowed by methy_CpG features during CART induction.

## Discussion

**Figure 3:**
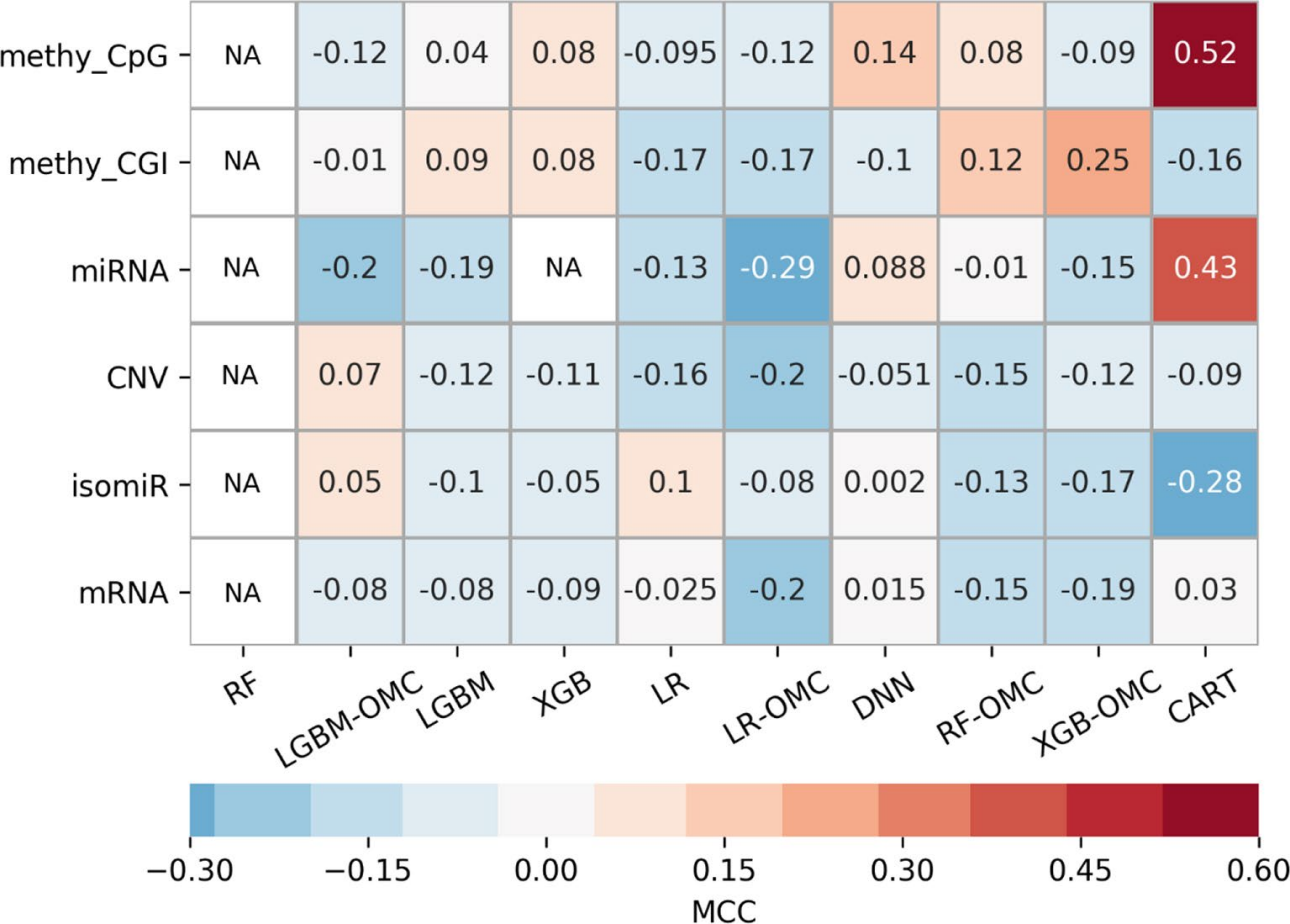
Employing multiple ML algorithms and tumor profiles increase the likelihood of discovering models able to predict BC patient response to paclitaxel. ML algorithms include the unaltered version of tree-ensemble and linear algorithms using all available features (RF, XGB, LGBM, and LR) and their OMC versions (RF-OMC, XGB-OMC, LGBM-OMC, and LR-OMC). The 9^th^ algorithm was CART, employed to generate simpler and more interpretable classification models. The 10^th^ algorithm was DNN, employed to generate more sophisticated but less interpretable models. Each of these algorithms was evaluated on each of the six molecular profiles, which resulted in 60 classifiers on the same BC patients. LOOCV evaluation was performed 5 times setting a different random seed for the employed ML algorithm, leading to 5 MCC determinations quantifying predictive performance. The heatmap shows the median MCC per classifier. Rows show the processed molecular profiles (‘CNV’ is short for copy-number variation, ‘methy_CpG’ for CpG site methylation, and ‘methy_CGI’ for CGI methylation), while columns display the employed ML algorithms. Thus, each cell corresponds to the median MCC of a given predictive model. Cells are colored in light-blue and dark-blue when this model reaches a negative or very negative median MCC (i.e. classification worse than random); in grey when it reaches a median MCC very close to 0.0 (i.e. random classification); in light-brown and dark-brown when it reaches a positive or very positive median MCC (i.e. classification better than random or close to perfect); in white and labelled NA (i.e. not available) when it reaches an undefined median MCC (i.e. misclassification of non-responders within several or all iterations). These results show that DNA methylation is the most informative profile (it leads to 2 of the 3 classifiers with a median MCC of a least 0.25). The choice of ML algorithm also affects the predictive performance. For example, none of the RF or LGBM classifiers obtain an MCC of at least 0.10. Thus, predictive performance depends strongly on of algorithm- profile combination: only one XGB-OMC models is predictive (that based on CGI methylation) and it is among the best predictors (median MCC of 0.25). Two other examples are the CART classifiers based on CpG methylation and miRNA expression, with median MCC of 0.52 and 0.43, respectively. **Figures S4** and **S5** further characterizes the performance of the best classifiers.

Owing to the wealth of curated data offered by the GDC, we could evaluate six profiles. The exhaustive evaluation of the 60 predictive models obtained, employing 10 ML algorithms with each profile, reveal strong variability in predictive performance (**Figure 3**). These results show the importance of considering multiple profiles and ML algorithms, the latter being always possible. For example, we could have carried out this study using the standard all-features versions of tree-ensemble, LR and DNN algorithms. However, this would have only resulted in models with near-random predictability despite using six profiles and thus, we could have concluded that precision oncology is not possible for paclitaxel-treated BC patients. Instead, we also tested algorithms generating models requiring only a handful of features (OMC-based and CART), which in addition, provided the best performance on these problem instances. Note that the most predictive of these models achieved an over 10,000-fold reduction in the number of features (**Table 1**).

Identifying a concise list of predictive molecular features is indeed beneficial for interpretability. The CGI methylation-based XGB-OMC model employs a dramatically reduced number of features (11 of the considered 11,644). The increased predictive performance comparing to all-features model (**Figure 1**) shows that the selected subset of features contains the information relevant for predictions (**Figure S2**). Therefore, applying OMC not only offers better predictivity, but also better interpretability of response to paclitaxel, as it revealed a molecular signature able to discriminate sensitive and resistant BC tumors from high-dimensional data. The best CART models reached the highest predictive performance among the generated predictors (**Figure 1**). Moreover, these models allow going further in the interpretation of response to paclitaxel (**Figure 2**). For example, the CpG-methylation DT unveils two rules employing only two features to predict which are the paclitaxel-sensitive BC tumors (**Figure 2**). The other example is the miRNA DT, which carries out these predictions using four induced rules based on only four features (**Figure 2**). Thus, the application of these rules to forthcoming tumors should improve paclitaxel treatment for BC patients. To facilitate such application, we are providing two python scripts in the supplementary materials, each implementing the rules for one of these predictive profiles.

Our best classifier obtained a median MCC of 0.54 in 10 LOOCV runs (an average MCC of 0.62, with MCC ranging from 0.48 to 0.87 in these runs as it can be seen in **Figure 1**). To put these predictive accuracies in the context of what is typically achieved when predicting tumor response to a drug from omics profiles, we have looked at other test set performances reported at the literature for this problem. One study (Kim et al., 2016) applied a range of ML algorithms to predict pancancer cell line response from transcriptomic profiles and obtained MCCs below 0.6 in all cases (see **Figure 1** in that paper). Maximum MCCs slightly above 0.5 and 0.3 were also obtained using RF with transcriptomic profiles (Nguyen et al., 2017) and genomic profiles (Naulaerts et al., 2017), respectively. Another study (Xu et al., 2019) also predicted drug response using many hundreds of pancancer cell lines using several ML algorithms from various omics profiles (gene expression, copy-number alterations, single-nucleotide mutations). Depending on the considered data resource, average MCCs across drug and profiles range from 0.15 to 0.31 or from 0.22 to 0.45 (see Tables 2 and 3 in that paper). Yet another example is by (Tripathi et al., 2016) using gene variants as features, where MCCs range across drugs from 0.32 to 0.56 or from 0.30 to 0.44 depending on data resource (see **Tables 1** and 2 in that paper). Lastly, single-gene drug response markers identified by MANOVA and Chi-Square tests on pancancer cell lines obtained maximum MCCs of 0.30 and 0.31, respectively (Dang et al., 2018).

The alteration of gene expression due to epigenetic modifications triggers the development of cancers, including BC. DNA methylation changes, occu rring both within and around CGIs, can impact transcriptional activity of genes or transcription factors involved in malignant phenotypes (Esteller, 2002; Irizarry et al., 2009; Levenson, 2010; Deaton and Bird, 2011; Manjegowda et al., 2017; Stirzaker et al., 2017). It has been shown that biomarkers for prognosis and treatment can be unearthed from DNA methylation profiles (Xiang et al., 2013; Mikeska and Craig, 2014; Stirzaker et al., 2014; Li et al., 2015; Pouliot et al., 2015). Furthermore, it has been found that DNA methylation can interfere in chemo-resistance to paclitaxel (Wang et al., 2012; Ignatov et al., 2014; Yun et al., 2015; He et al., 2016; Zhang et al., 2018). Our DNA-methylation-based predictors selected CpG sites and CGIs related to genes previously found individually involved in cancer development and with transcriptional activity regulated by methylation (e.g. MBTPS2, YY2, ECRG4, IKZF1). Selected features by these models are also related to genes associated to response to cytotoxic drugs such as N4BP2L2 (paclitaxel), CYP2D6 (tamoxifen), APOBEC4 (tamoxifen, doxorubicin, and etoposide), and TUBB8 (paclitaxel) (**Table S15**).

miRNAs also play a key role in cancer development by acting as tumor suppressors or oncogenes. These molecules can be used as biomarkers, and modulation of their specific activities provides insight for therapeutic investigations (Hayes et al., 2014; Peng and Croce, 2016). Furthermore, the expression of some miRNAs has been associated to the sensitivity to paclitaxel (Zhou et al., 2010; Chen et al., 2014; He et al., 2016; Lu et al., 2017). The miRNA expression-based CART model combines miR-27a-3p, miR-217, miR-200c-5p, and miR-942-5p to predict which BC tumors are paclitaxel-responsive with high accuracy (**Figures 1** and **2A**). Individually, each of these miRNAs have been linked to paclitaxel response and BC prognosis: the first three are related to paclitaxel resistance, whereas the last one is associated to shorter survival of BC patients (**Table S15**).

Our study has some limitations to be pointed out. First, for a given patient, molecular profiles were obtained from the primary tumor, while clinical response was registered later following tumor evolution. Both tumors may present some differences at the molecular level, due to temporal or spatial tumor heterogeneity, as well the possible impact of the treatment administered after tumor resection. Second, while we reported predictive accuracy on BC tumors not used in any way to build or select the model, an additional independent evaluation on a second cohort would shed further light into how general these models are. The latter is currently not possible due to the scarcity of paclitaxel-treated BC patients with DNA methylation or miRNA profiles of their tumors.

**Figure 4:**
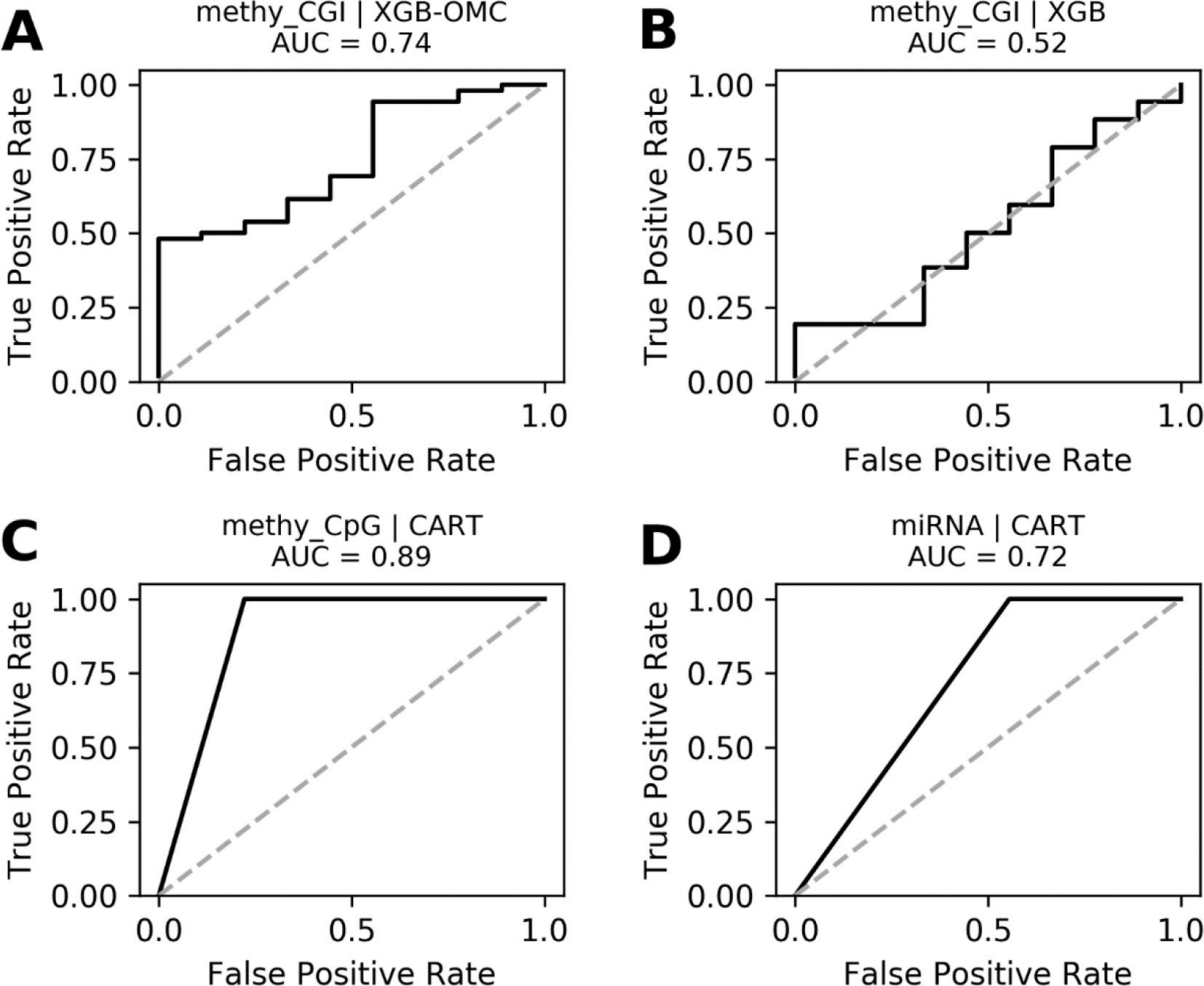
ROC curves of the most predictive case of best models. ROC curves obtained plotting the true positive rates against the false positive rates calculated from the models presented in **Figures S4**. The AUCs were calculated from the predictions that came out from the nested and standard LOOCV runs and were respectively carried out for OMC and CART models. We notice that AUCs follow the same trend as MCCs and that models shown in **(A)**, **(C)**, and **(D)** are very robust. The dashed line delimitates the expected AUC from random classification.

Yet, our work provides very predictive (in the context of the considered problem), robust (**Figure S4** and **Figure 4**), and even interpretable models to identify primary BC tumors sensitive to paclitaxel. These results also suggest that tumor methylomes and miRNomes can be a source of multi-variate models to predict prognosis and treatment response. Indeed, our predictive models reveal molecular features that can collectively anticipate which BC tumors are sensitive or resistant to paclitaxel. Previous studies have experimentally validated the involvement in BC development, and even in the resistance to paclitaxel, of these molecular factors individually, which further supports the applicability of these classifiers. Furthermore, our results also suggest novel predictive factors such as the antisense to MRGPRX4; the pseudogenes (Poliseno et al., 2015; Xiao-Jie et al., 2015) C6orf108 and FAM166A; and the coding genes NDUFA6-AS1, UXS1, RGL1, and LEXM.

## Data Availability Statement

The datasets generated for this study can be found in the https://portal.gdc.cancer.gov/.

## Author Contributions

PB conceived the study and designed the experiments. AB and PB wrote the manuscript with the assistance of AG. AB carried out the numerical experiments. All authors analyzed the results and contributed to their discussion.

## Funding

This work was supported by the Institut Paoli-Calmettes (grant number 305/2016 to PB).

## Conflict of Interest

The authors declare that the research was conducted in the absence of any commercial or financial relationships that could be construed as a potential conflict of interest.
