# Supplementary material for: Paclitaxel Response Can Be Predicted With Interpretable Multi-Variate Classifiers Exploiting DNA-Methylation and miRNA Data
Source: Front Genet. 2019 Oct 25;10:1041. doi: 10.3389/fgene.2019.01041 (PMC6823251; doi:10.3389/fgene.2019.01041)
Supplement: Supplementary file 1 [file DataSheet_1.doc]

**Paclitaxel response can be predicted with interpretable multi-variate classifiers exploiting DNA-methylation and miRNA data**

Alexandra Bomane, Anthony Gonçalves and Pedro J. Ballester^*^

Cancer Research Center of Marseille, CRCM, INSERM, Institut Paoli-Calmettes, Aix-Marseille Univ, CNRS, F-13009 Marseille, France.

* correspondence to: Pedro Ballester, email: pedro.ballester@inserm.fr

**SUPPLEMENTARY INFORMATION**

**SUPPLEMENTARY METHODS**

**Processing clinical data for modelling**

Paclitaxel synonyms found in clinical data were manually standardised to correct heterogeneous annotations (Supplementary Table S9).

**Molecular profiles for modelling**

To build miRNA and isomiR expressions datasets, we applied the procedure described in the documentation of the miRNA profiling pipeline developed by the British Columbia Genome Sciences Centre (Chu et al., 2016). Then, the expression of mature miRNAs and the one of isomiRs, that are defined by their coordinates (Supplementary Table S10 reports the index of the newly defined features), were obtained. In this way, we are able to distinguish the actual expression of mature miRNA from the one of precursor miRNA (Cai et al., 2009; Luciano et al., 2004), and the actual expression of isomiRs that could occur in tumours (Guo et al., 2018; Liao et al., 2018; Telonis et al., 2015). miRNA and isomiR expressions are log2-transformed RPMs (reads per million mapped reads).

To analyse DNA copy number data generated by microarrays, the Genomic Data Commons (GDC) applied a workflow using Circular Binary Segmentation (CBS) algorithm (Olshen et al., 2004) from the *DNAcopy* R package (Seshan and Olshen, 2014) to identify altered chromosome segments within each sample in order to find features defined in all samples. Samples usually differ in the number of altered regions and even for the same region where multiple samples show aberrations the margins of the altered region rarely align across samples. Thus, the output of CBS cannot be arranged as a matrix on which most computational algorithms operate. As the result, ML analysis cannot be applied directly to segmented DNA copy number data. To overcome this issue, we used the *CNTools* R package version 1.33.0 (Jianhua, 2018) which provides the functionalities for converting the segmented DNA copy number data into a matrix format to facilitate further computational analyses matching segments to their corresponding gene(s), whose copy numbers constitute the features of the dataset. To deal with genes having multiple segment values, we assign their average DNA copy number.

The GDC’s pipeline uses data from the Illumina Infinium Human Methylation 27 (HM27) and Human Methylation 450 (HM450) arrays to measure the level of methylation at known CpG sites as beta values. Beta value represents the ratio between the methylated array intensity and total array intensity, it falls between 0 (lowest level of methylation) and 1 (highest level of methylation). HM27 and HM450 probes were remapped to build 38 of the reference genome provided by Gencode (GDC Reference Files | NCI Genomic Data Commons) (Supplementary Table S7). These coordinates were then used to identify the associated transcripts, the associated CpG Island (CGI), and the CpG sites’ distance to each of these features. First, we produced a methylation beta value dataset where probes constitute the molecular features. Second, we generated a dataset where methylation beta values of CpG sites were averaged by the coordinates of the CGI they are associated with (Supplementary Table S8 reports the index of the newly defined features). This allows to make a distinction between the methylation level of individual CpG sites represented by probes and the one of CpG sites occurring within CGIs and around. mRNA expression is FPKM-normalized (Fragments Per Kilobase Million).

**Processed pharmaco-omic datasets for modelling**

Supplementary Table S3 summarises the number of data instances and features contained by each dataset. Only those features which constitute these datasets are defined in the all data instances to avoid missing values. The drug response of each data instance was annotated as “NonResponder” or “Responder“, respectively encoded as 0 and 1 prior to ML.

**Measuring the predictive performance of a classifier**

A pharmaco-omic dataset can be represented as:

$$Data=\left\{\left(class^{\left(k\right)},x^{\left(k\right)}\right)\right\}_{k=1}^{k=n}$$

Where *x* is a high-dimensional vector with the molecular features from the considered profile for the *n* breast cancer (BC) patients’ tumours that have been treated with paclitaxel. In ML field, dealing with two possible predicted classes is called binary classification problem. Positive data instances are tumours sensitive to paclitaxel (*class* = responder), whereas negatives are resistant tumours (*class* = non-responder).

Each dataset constitutes an input for ML algorithms in order to train classifiers which are able to predict response to paclitaxel. Ideally, these classifiers should be trained on our pharmaco-omic datasets and then evaluated on an independent dataset. However, the limitation of available data drives to employ cross-validation, which is a model validation technique for assessing how much the model is able to generalize to an unseen dataset (Kohavi, 1995). As shown in Supplementary Table S3, datasets are made of a small number of tumours, as it is typical in this problem. Consequently, we choose to evaluate the predictive performance of all-features classifiers with standard Leave-One-Out Cross-Validation (LOOCV). Moreover, nested LOOCV is used for selecting and evaluating classifiers employing Optimal Model Complexity (OMC) (Nguyen et al., 2018) to avoid overestimating their predictive performance (Cawley and Talbot, 2010; Varma and Simon, 2006). Actually, nested LOOCV is nothing but a standard LOOCV where the model optimised with the training set of a given fold is applied to the corresponding held-out sample of that fold. This means that optimized models are trained and selected without the information from the held-out samples.

Once known and predicted classes are compared for all held-out samples, we count the true positives (TP), true negatives (TN), false positives (FP) and false negatives (FN) among BC patients’ tumours as described in the contingency table showed in Table 1. Thus, these counts are used for calculating some classification metrics which summarize the predictive performance reached by a classifier.

Precision (PRE) and recall (REC) (Van Rijsbergen, 1979) represent the general characteristics of the classifier. PRE and REC are expressed as follow:

$$PRE=\frac{TP}{TP+FP}$$

$$REC=\frac{TP}{TP+FN}$$

In this study, PRE reflects the ability of the classifier to label as *responder* samples that are indeed responsive (i.e. PRE value of 0 means that all tumour samples predicted *responder* are actually *non-responder*, while PRE value of 1 indicates that all the tumour samples predicted *responder* are effectively found to be *responder*). REC (or true positive rate), reflects the ability of the classifier to correctly identify responders (i.e. REC is 0 if all responsive tumours are misclassified, whereas REC is 1 if all responsive tumours are correctly predicted).

F1-score (F1) (Van Rijsbergen, 1979) is the harmonic average of PRE and REC. F1 is calculated by the following formula:

$$F1=\frac{2.PRE.REC}{PRE+REC}$$

F1 reaches its best value at 1 when perfect REC and REC (thus both have a value of one), and its worst at 0 when null PRE and/or REC (i.e. responders are never correctly predicted). PRE, REC and F1 were computed using the functions *precision_score()*, *recall_score()* and *f1_score()* provided by *Scikit-Learn* (Pedregosa et al., 2011) setting the parameter *average* as “weighted” to account for label imbalance.

Furthermore, Supplementary Table S3 also shows that responsive tumours outnumber non-responsive tumours. Thus, it is important to consider a classification metric which measures the ability of the classifier to identify both the majority class (*responder*) and the minority class (*non-responder*). Matthews Correlation Coefficient (MCC) is a measure of the quality of binary classifications (Matthews, 1975). This metric takes into account true and false positives and negatives and is generally regarded as a balanced measure which can be used even if the classes are of very different sizes (Boughorbel et al., 2017). MCC is defined as follows:

$$MCC=\frac{TP.TN-FP.FN}{\sqrt{\left(TP+FN\right).\left(FN+TN\right).\left(TN+FP\right).\left(FP+TP\right)}}$$

MCC can take values from -1 to 1, where 1 means that the classifier provides perfect agreement between observed and predicted classes (i.e. both responders and non-responders are perfectly classified), -1 indicates a perfect disagreement (i.e. all responders are predicted as non-responders, and vice-versa) and 0 means that the classifier predicts the class randomly. If any of the two classes is not predicted at all, MCC will be undefined. MCC was computed using a modified version of the function *matthews_corrcoef()* provided by *Scikit-Learn* which returns ‘NaN’ (“Not a Number”) instead of zero, in order to make the distinction between zero-valued and undefined MCCs. It has been shown that chi-square statistic can also summarize the predictive performance of a classifier (Dang et al., 2018).

Indeed, MCC is related to chi-square statistic for a 2 × 2 contingency table

$$\left(MCC\right)=\sqrt{\frac{\chi^{2}}{n}}$$

The formula to compute the chi-square statistic is

$$\chi^{2}=\sum_{i=1}^{2} \sum_{j=1}^{2} \frac{\left(O_{ij}-E_{ij}\right)^{2}}{E_{ij}}$$

where *i* and *j* represent respectively rows and columns in the contingency table, O*ij* are the frequency counts (TP, TN, FN, FP) and E*_ij_* are the corresponding expected values under the null hypothesis that this partition has arisen by chance. Thus, expected values are calculated with

$$E_{11}=E\left(TP\right)=PP.\frac{OP}{n}$$

$$E_{12}=E\left(FN\right)=PN.\frac{OP}{n}$$

$$E_{21}=E\left(FP\right)=PP.\frac{ON}{n}$$

$$E_{22}=E\left(TN\right)=PN.\frac{ON}{n}$$

For instance, the expected value of TP, E(TP), is the number of predicted positives (PP) times the probability of a tumour being a positive given as the proportion of observed positives (OP) in the *n* tumours.

This Pearson’s chi-square test statistic follows a χ² distribution with one degree of freedom and thus each *p*-value was computed with the function *chi2_contingency()* from the python library *Scipy* version 1.0.1 (with the parameter *correction* set as “False”) using the contingency tables provided in Supplementary Table S5.

The Receiver Operating Characteristics (ROC) curve shows the tradeoff between the true positive rate and the false positive rate (Fawcett, 2006). Such plot provides a model-wide evaluation of binary classifiers. The Area Under the Curve (AUC) score is a valid measure of the ROC curve. AUC is 0.5 for random and 1 for perfect classifiers (Hanley and McNeil, 1982). AUC scores are convenient to compare the performances of multiple classifiers. This metric was computed using the functions *roc_curve()* and *auc()* provided by *Scikit-Learn*.

**Predicting drug response using ML algorithms with embedded feature selection**

Classification And Regression Tree (CART) (Breiman et al., 1984) grows a Decision Tree (DT) operating binary splitting of training data instances based on conditions assessed on the most informative features that were internally selected. Thus, it allows discovering interactions among independent features leading to the predictions. CART models were built using default parameters. These parameters can be found at http://scikit-learn.org/stable/modules/generated/sklearn.tree.DecisionTreeClassifier.html. By default, this implementation does not have a deterministic behaviour. The default parameter *splitter* set as “best” allows employing an algorithm which randomly chooses one of the features and calculates its best possible data split using the Gini impurity metric. This task is repeated as many times as features are considered (Liu et al., 2005). In addition, the features are presented in a randomly permuted order at each split. Therefore, the best split found may vary, even with the same training data and *max_features* equals to all features, if the improvement of the Gini impurity is identical for several splits enumerated during the search of the best split.

Random Forest (RF) (Breiman, 2001) models combine several DTs and uses the bootstrap aggregation (or “bagging”) (Breiman, 1996) to reduce variance of predictions. Default values for the hyperparameters of the employed implementation can be found at http://scikit-learn.org/stable/modules/generated/sklearn.ensemble.RandomForestClassifier.html. These values were used in our RF models, except for that of the number of trees in the forest (*n_estimators*). It has been shown that the number of trees should only be chosen to be sufficiently large for the Out-Of-Bag (OOB) error to have stabilised (Svetnik et al., 2003). In many cases 500 trees are sufficient for this purpose and there is no penalty for having too many trees other than waste in computational resources (i.e. no further reduction of error is achieved with RF if more than 500 trees are used (Svetnik et al., 2003)). As a preclinical instance of this problem achieved predictive models using RF with 1000 trees (Xu et al., 2019), we also set *n_estimators* to this value.

In contrast, models produced by gradient boosting algorithms (Friedman, 2001, 2002) grow DTs sequentially (i.e. each tree is grown using information from previously grown trees). XGBoost (XGB) (Chen and Guestrin, 2016) and LightGBM (LGBM) (Ke et al., 2017) are two of this type of algorithm. The main difference between them is that LGBM uses a histogram-based algorithm to speed up the training process, reduce memory consumption, and adopt a leaf-wise growth strategy with depth limitation. Default parameters of XGB can be found at https://github.com/dmlc/xgboost/blob/v0.60/python-package/xgboost/sklearn.py.

Default parameters of LGBM can be found at https://github.com/Microsoft/LightGBM/blob/v2.0.10/python-package/lightgbm/sklearn.py. For both XGB and LGBM algorithms, *n_estimators* was set to 1000, and both the subsample ratio of data instances (*subsample*) and subsample ratio of features (*colsample_bytree*) were set to 0.8 to enable the bootstrap aggregation.

Deep Neural Network (DNN) (Bengio, 2009) algorithms seek mathematical operations to turn the input (features) into the output (variable to predict) by modelling this relationship. Each mathematical operations is a layer here, with a DNN having at least two layers, thus the adjective "deep". DNN, in general, have many tunable hyperparameters. However, recent studies (Zhou et al., 2019) report sets of hyperparameter values providing consistently good performance across a high number of drug design-related tasks: the authors have performed a grid search over several hyperparameters and found setups that gave consistently high performance for 17 ADME-related classification tasks. Therefore, we apply here the top setup proposed in this work. The neural network consists of an input layer (the number of neurons is equal to the number of features of the training set), three hidden layers with 512, 256, and 64 neurons, consecutively, and the output layer of one neuron. Activation function of all neurons in the hidden layers is Sigmoid (Han and Moraga, 1995). Adam optimizer (Kingma and Ba, 2014) with the learning rate (*lr*) equal to 1.0 is used. To reduce overfitting, dropout (Srivastava et al., 2014) of 0.6 and weight decay (*kernel_regularizer*) (Loshchilov and Hutter, 2017) of 10^-6^ is applied to all hidden layers. Additionally, the employed loss function (*loss*) is binary cross-entropy (Murphy, 2012) and training lasted for 100 epochs for any model.

Logistic Regression (LR) (Ranstam et al., 2016) models simply use linear combinations of one or more independent variables to model probability output in terms of input. Default parameters of LR can be found at https://scikit-learn.org/stable/modules/generated/sklearn.linear_model.LogisticRegression.html .

In this study, when all features available in a processed dataset are considered during model training and the default operating threshold (0.5) is used, we are dealing with an “all-features model”. Such classifier is evaluated with standard LOOCV, repeated setting several different random seeds to assess the variability introduced by the stochastic character of ML algorithms. As shown in Supplementary Table S3, responders outnumber non-responders. To mitigate unbalanced datasets issue, sample weighting was applied during model fitting. To this end, the function *compute_sample_weight* from *Scikit-Learn* with the parameter *class_weight* set as “balanced” allowed to calculate sample weights.. Thus, weights are adjusted inversely proportional to class frequencies in the input data so that the instances belonging to the minority class get larger weights (i.e. higher misclassification cost). These weights are then given to the parameter *sample_weight* of the function *fit* of the trained classifier. Class-permutation test was performed to see the significance of predictive performances obtained from standard LOOCVs. This test consists in shuffling the labels contained in a considered training set applying LOOCV, resulting in estimations of the predictive performance reached by a “permutation model”. The *p*-value assessing the significance of the difference between the scores obtained by all-features models and permutation models comes from two-sided paired Student’s *t*-test (function *ttest_rel* from the *Scipy* python library).

**Predicting drug response using OMC**

An effective way to improve predictive performance is to reduce the dimensionality of the data. Here, data dimensionality can be defined as the number of considered features over the number of patients’ tumours. One route to reduce dimensionality is hence to use more training data, but these are usually not available. An alternative route is to only consider the most informative features in the data discarding the irrelevant ones (hence strongly reducing data dimensionality while retaining most the initial information content). We designed OMC as a strategy to build ML models employing only the most relevant features.

Let us consider a standard LOOCV resulting in an outer training fold and a left-out sample. On a given outer training fold made of *N* samples and *M* features, a second LOOCV is performed resulting in an inner training fold and a left-out sample: this corresponds to a nested LOOCV. On each inner training fold, the following operations are made:

1) *M* *p*-values, one per each of *M* features, are calculated to measure the discriminative power of each feature regarding drug response (*t*-test).

2) All features are ranked by increasing *p*-value (i.e. the most informative features are those with the smallest *p*-values).

3) From this ranking, models with top-2 to top-*N*/2 features are considered.

4) For each model complexity,

a) the model is trained on the inner training fold.

b) This model is employed on the corresponding inner test fold to calculate class probabilities.

c) Cutoffs going from 0.0 to 1.0 (with a step of 0.05) are tried as operating thresholds to assign class label(s) of the corresponding inner test fold, based on the class probabilities previously obtained.

d) An MCC is calculated for each corresponding cutoff.

e) The cutoff leading to the highest MCC is set as the operating threshold for the current model complexity.

f) AUC of the inner test fold is calculated using the current complexity and the corresponding optimal operating threshold.

5) The model which achieves the highest AUC is selected.

6) This model is trained with all instances comprise in the current inner training fold

7) This optimal model (tuned complexity and operating threshold) is applied to predict the class label of the corresponding outer test fold.

It is noteworthy to highlight that this process allows to tune both the model complexity and the operating threshold separately from unseen data that are used for the model evaluation. Supplementary Figure S1 illustrates the process. In this study, predictive models obtained using an ML algorithm completed with OMC is called “OMC model”. To see the significance of predictive performances obtained by these models, their scores are compared with the ones reached by permutation models as described in the previous section. As CART algorithm is already able to reduce model complexity selecting a small subset of features, we did not apply OMC with CART models.


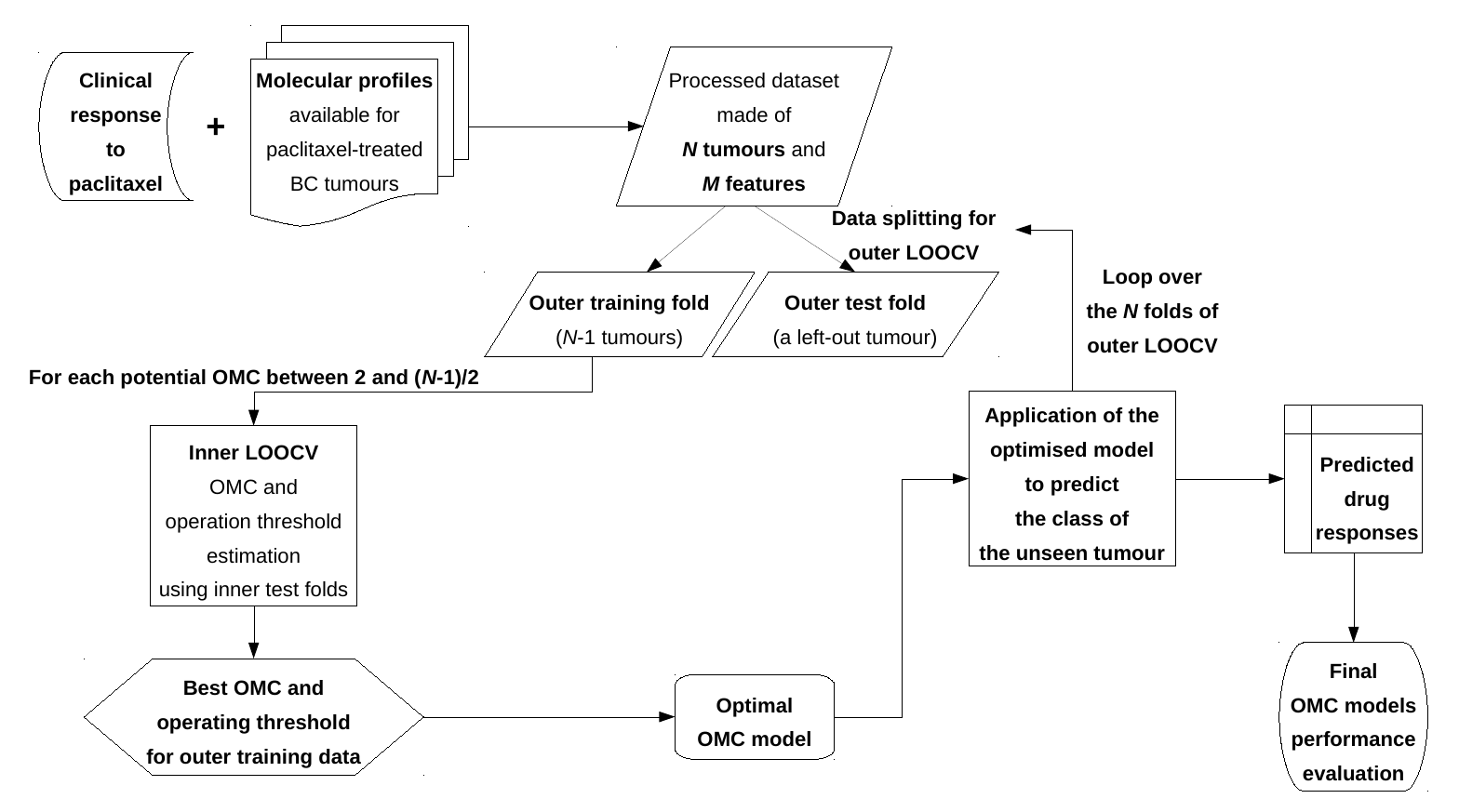


**Supplementary Figure S1: Workflow to evaluate the predictive performance of models using the Optimal Model Complexity (OMC).** An OMC model is obtained tuning both the complexity and the operating threshold during its training, so that it considers only the selected features and use the most suitable operating threshold for predicting drug response of BC tumours. Such model is evaluated performing a nested LOOCV. In this way, each tumour is ultimately predicted with an optimised model that was tuned and trained on the rest of tumours.

**Computing averaged ranking of features**

RF uses Gini impurity as objective function, while both XGB and LGBM employ logistic loss (cross-entropy loss). The importance of a feature is computed as the normalized (i.e. values range from 0.0 to 1.0) total reduction of the applied criterion brought by this feature. Then, features were ranked by increasing importance.

**RESULTS**

**High-dimensional data may cause the poor predictive performance of all-features ML models (RF and XGB), while the best complexity-optimized ML models (RF-OMC and XGB-OMC) give a better predictive performance because they employ a much smaller subset of informative features**

GDC datasets are of high to very high dimensionality. There are typically several molecular profiles available for each patient, each containing from several hundred to many thousands of features. Processing these datasets, we noticed that molecular features always far outnumber the patients’ samples, which leads to high-dimensional data and can impact the ability of models to classify data instances. Such issue is very common in ML applied to bioinformatics field (Saeys et al., 2007) and tends to induce model overfitting, challenging its predictive performance. A solution to deal with this problem is to select the most informative features to build the model. Estimating the OMC (Nguyen et al., 2018) is a strategy for data-driven identification of the subset of most predictive features. This procedure results in models that ignore many thousands of irrelevant features. In using OMC, we expect to mitigate the effect of overfitting and hence increase the predictive performance of ML models (i.e. reduce both false positives and false negatives) focusing on the most important features.

Let us consider the case of methylation-based models with improved predictive performance using OMC and their equivalents which consider all features.

Supplementary Figure S2 shows that the number of features considered by all-features models during model training far outnumbers the ones considered by OMC models. Indeed, CpG site methylation-based all-features models consider 22,941 features, while CGI methylation-based ones consider 11,644 features. In contrast, CpG site methylation-based OMC models consider 26 to 29 features, while CGI methylation-based ones consider 11 to 30 features. Thus, dealing high-dimensional data, all-features models tend to overfit training data more, which impacts their ability to generalize on unseen instances and leads to poor predictive performance. Moreover, we can also see that all-features models actually use very few features for predictions. Indeed, over all these models, among all features available in datasets 1% to 20% of features participate in predictions. For instance, CGI methylation-based RF models use 2,329/11,644 features (20%) for predictions and give undefined MCCs as they are not able to predict non-responders. This not only means that a high proportion of features is ignored by the predictive models to make predictions, but also that the operated feature selection is not sufficient to find the features which are informative in the context of the problem. Thus, high-dimensional data interferes with the ability of all-features models to extract the relevant features. Furthermore, the CGI methylation-based XGB model considers only 1% of features as informative because of tree pruning internally performed by the algorithm. This process is controlled by the maximum delta step allowing each tree’s weight estimation to be and the minimum sum of instance weight needed in a leaf (*max_delta_step* and *min_child_weight*) for XGB.

Supplementary Figure 2B shows that using OMC leads to focus on small subsets of features which are actually used to predict response to paclitaxel. Indeed, over all the OMC models, the percentages of features actually used in predictions go from 70% to 100%. For instance, CGI methylation-based RF-OMC models use the totality of features considered during model training (11 to 29) for predictions and give a better predictive performance than the models which consider the 11,644 features available. This means, that not only OMC allows to reduce the complexity of models, but also it helps to perform model training with features which are informative in the context of the problem. Thus, integrating ML algorithms with OMC can increase predictive performance by restricting to an optimal subset of features. When looking at these features individually, Supplementary Figure S3 shows that the level of methylation tends to be higher for responders than for non-responders

| Tumour profiling data | ML algorithm | Number of considered features | Median OMC | Median optimal operating threshold | Median MCC  All-features models | Median MCC  OMC models | *p*-value  OMC MCCs vs permutation MCCs | *p*-value  OMC MCCs vs all-features MCCs |
| --- | --- | --- | --- | --- | --- | --- | --- | --- |
| methy_CpG | RF | 22,941 | 28 | 0.50 | NaN | 0.15 | 3.67∙10^-4^ | 3.67∙10^-4^ |
| methy_CGI | RF | 11,644 | 12 | 0.65 | NaN | 0.13 | 1.21∙10^-3^ | 1.21∙10^-3^ |
|  | XGB |  | 11 | 0.05 | 0.08 | 0.25 | 9.30∙10^-4^ | 2.16∙10^-2^ |

**Supplementary Table S12: Performance of the best methylation-based OMC models.** The predictive performance of these models is seen in Figure 1B. OMC models were obtained being trained on a given dataset considering all data instances and selected features. The median MCCs were obtained from the 10 iterations of standard LOOCV for all-features models, and permutated LOOCV for permutation models. The comparison of MCC scores obtained by OMC models with the ones obtained by all-features and permutation models (class-permutation test) is quantified via a p-value calculated from two-sided paired Student’s t-test. The first model was trained on CpG methylation (shorten as ‘methy_CpG’) employing RF-OMC: it retained 28 out of 22,941 features and applied an operating threshold of 0.50 reaching a median MCC of 0.15 and performing significantly better than permutation and all-features models (p-values equal = 3.67∙10^-4^). The second model processed CGI methylation (shorten as ‘methy_CGI’) employing RF-OMC: it retained 12 out of 11,644 features and applied an operating threshold of 0.65 achieving a median MCC of 0.13 and performing significantly better than permutation and all-features models (p-values equal 1.21∙10^-3^). The last model was also based on CGI methylation and employed XGB-OMC: it retained 11 out of 11,644 features and applied an operating threshold of 0.05 returning a median MCC of 0.25 and performing significantly better than permutation (p-value = 9.30∙10^-4^) and all-features (p-value = 2.16∙10^-2^) models. Predictive performance resulting in undefined MCC is indicated with ‘NaN’ (“Not A Number”): NaNs were set to zero to be able to perform the statistical test.

| methy_CGI-XGB | | methy_CGI-RF | |
| --- | --- | --- | --- |
| Feature | Averaged ranking | Feature | Averaged ranking |
| CGI_ID.24217* | 1.2 | CGI_ID.24217* | 1.0 |
| CGI_ID.15915* | 5.1 | CGI_ID.16043* | 2.7 |
| CGI_ID.6919* | 5.1 | CGI_ID.5459* | 3.2 |
| CGI_ID.5276 | 6.2 | CGI_ID.15915* | 4.2 |
| CGI_ID.5459* | 8.0 | CGI_ID.6919* | 5.5 |
| CGI_ID.16043* | 8.8 | CGI_ID.11106 | 7.4 |
| CGI_ID.11903 | 9.3 | CGI_ID.13390 | 8.8 |
|  |  | CGI_ID.21722 | 9.6 |
|  |  | CGI_ID.3533 | 9.8 |

**Supplementary Table S13**: **Most informative features from the best CGI methylation-based OMC models.** OMC models were obtained being trained on the CGI methylation dataset considering all data instances and selected features setting 10 different random seeds, and then feature importance were computed. This table reports the features with averaged ranking higher or equal to 10.0 from the rankings on feature importance returned by the 10 built OMC models. Cells with ‘*’ indicate features that are common to OMC models employing XGB and RF. ‘methy_CGI-XGB’ and ‘methy_CGI-RF’ are short for CGI methylation dataset processed by XGB and RF, respectively. The importance and averaged ranking of features selected by these OMC models are reported in Supplementary Table S11.

| CGI coordinates identifier | Related gene(s) |
| --- | --- |
| CGI_ID.24217 | CYP2D6, NDUFA6-AS1, RP4-669P10.19 |
| CGI_ID.15915 | MBTPS2, YY2 |
| CGI_ID.6919 | C2orf40, UXS1 |
| CGI_ID.5276 | IKZF1 |
| CGI_ID.5459 | APOBEC4, RGL1, ARPC5, NCF2, SMG7 |
| CGI_ID.16043 | C1orf177 |
| CGI_ID.11903 | RP11-631M21.6, TUBB8 |

**Supplementary Table S14**: **CGI coordinates selected by CGI methylation-based XGB models using OMC mapped to related genes.** This table maps CGI coordinates selected by CGI methylation-based XGB model using OMC reported in Supplementary Table S12 with their related genes. The indices of CpG probes and CGI coordinated identifiers are respectively provided in Supplementary Tables S7 and S8. First, the index of CGI coordinates identifiers is consulted: for a given identifier (in column “CGI_ID”), its corresponding coordinates are found from the column “CGI_Coordinate”. Then, the index CpG probes is consulted: entries of probes located within and near the considered CGI are identified using these coordinates in the column “CGI_Coordinate”. Finally, for each entry, gene(s) related to a given probe is/are found from the column “Gene_Symbol”.


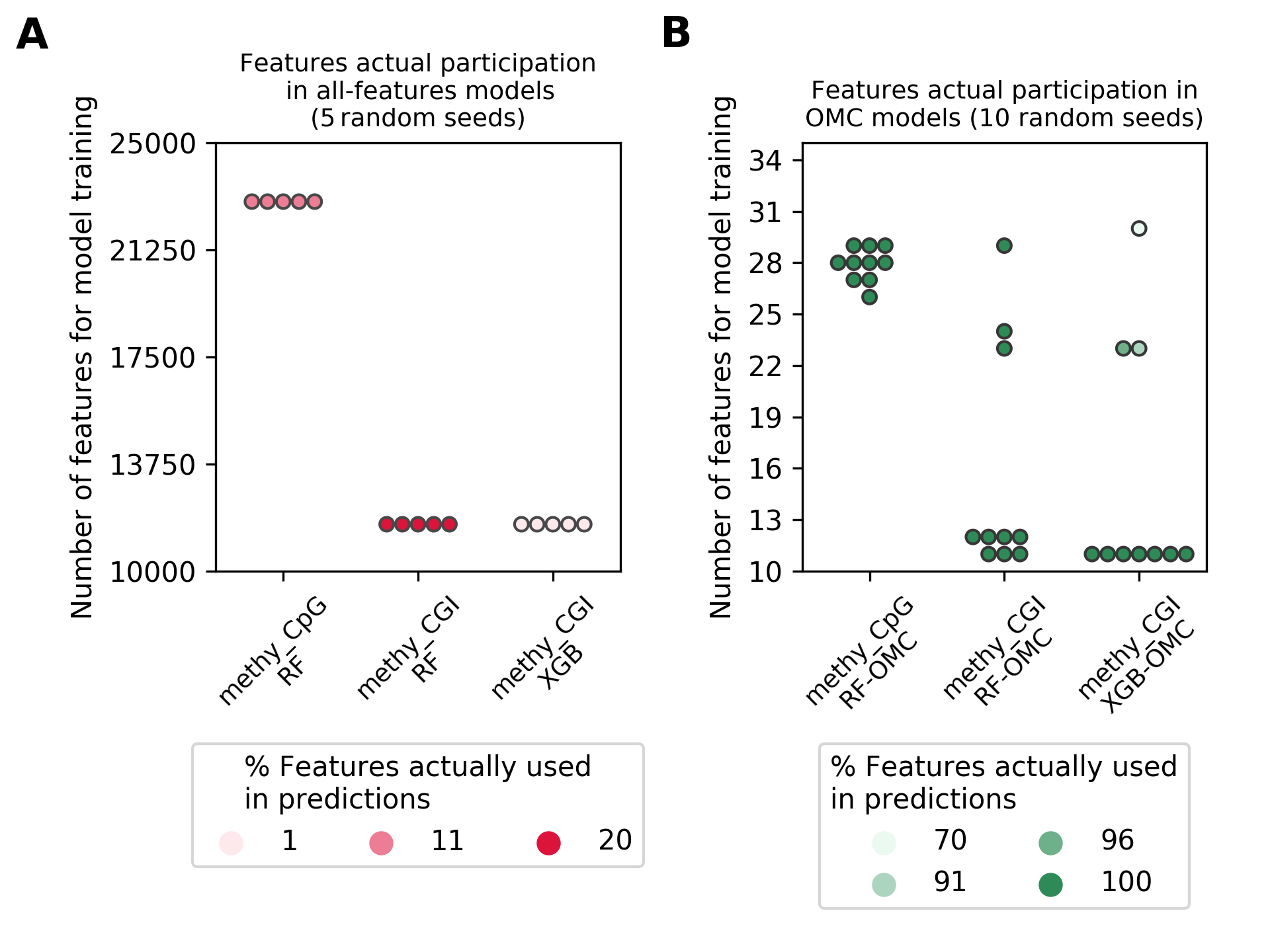
**Supplementary Figure S2**: **Comparison of the actual participation of features in predictions made by the methylation-based models improved using OMC with the ones considering all features.** A feature actually used in predictions made by a classifier reaches a feature importance higher than zero. These swarmplots show the proportion of such features out of the totality of features considered during model training from both all-features and OMC models. **(A) All-features models** were trained on a full given dataset (i.e. considering all features and data instances) and then feature importances were computed. This process has been carried out setting five different random seeds. *x*-axis shows processed datatypes along with the employed ML algorithm, while *y*-axis displays the number of features considered during model training: for all-features models, it corresponds to the number of features contained in a dataset. Each dot represents the percentage of features actually used in predictions made by all-features models among all features available in the processed dataset. The darker it is, the higher is the percentage: in this case, a deep red dot means that among all features, 20% are actually used in predictions, while a light red dot means that among all features, 1% are actually used in predictions. **(B)** **OMC models** were obtained being trained on a given dataset considering all data instances and optimal number of features and then feature importances were computed. This process has been carried out setting 10 different random seeds. *x*-axis shows processed datatypes along with the employed ML algorithm completed with OMC, while *y*-axis displays the number of features considered during model training: for OMC models, it corresponds to the optimal number of features selected from the processed dataset. Each dot represents the percentage of features actually used in predictions made by OMC models among features selected by the OMC from the processed dataset. The darker it is, the higher is the percentage: in this case, a deep green dot means that among features selected by the OMC, 100% of features are actually used in predictions, while a light red dot means that among features selected by the OMC, 70% of features are actually used in predictions. ‘methy_CpG’ is short for CpG methylation and ‘methy_CGI’ for CGI methylation.

**Optimization of complexity and operating threshold leads to better performance for some methylation-based OMC models than when all features are considered (Figure 1B and Supplementary Table S6)**

CpG site methylation-based RF-OMC models reach a median MCC of 0.15 and perform significantly better than both permutated RF-OMC and non-permutated all-features models (*p*-values from two-sided paired Student’s *t*-test equal 3.67∙10^-4^ in both cases, as all MCCs are undefined). Fitting such models on the dataset considering all data instances and optimal number of features and setting 10 different random seeds identifies a median of 28 out of the 22,941 CpG sites as the most informative features to predict response to paclitaxel. It is noteworthy that as the median optimal operating threshold equals the same as default (i.e. 0.50), we can deduce that the improvement of the predictive performance is solely due to the decrease of the model complexity.

CGI methylation-based RF-OMC models achieve a median MCC of 0.13 and perform significantly better than both permutation and all-features models (*p*-values equal 1.21∙10^-3^ in both cases, due to undefined MCCs). Fitting such models on the dataset as described previously and setting 10 different random seeds leads to a median of 12 out of the 11,644 CGIs as the most informative features and a median optimal operating threshold of 0.65.

CGI methylation-based XGB-OMC models give a median MCC of 0.25 and performs significantly better than both permutation and all-features models (*p*-values equal 9.30∙10^-4^ and 2.16∙10^-2^, respectively). Fitting such models on the dataset as described previously and setting 10 different random seeds returns a median of 11 out of the 11,644 CGIs as the most informative features and a median optimal operating threshold of 0.05.


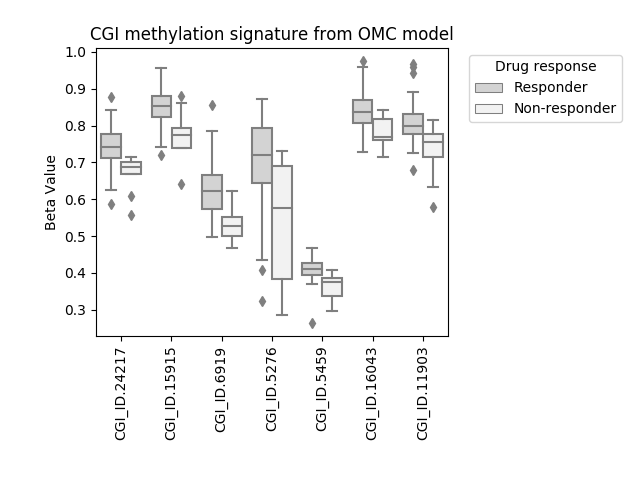
**FIGURES**

**Supplementary Figure S3**: **CGI methylation signature in resistant and sensitive paclitaxel-treated BC patients extracted by OMC models.** Features were extracted considering the highest averaged rankings on feature importances (Supplementary Table S14): this led to seven informative features. *x*-axis shows the encodings of CGI coordinates, while *y*-axis displays beta values associated to CGIs. Grey and light-grey boxplots summarise the distributions of beta values associated to responders and non-responders, respectively. Overall, the level of methylation tends to be higher for responders than for non-responders.


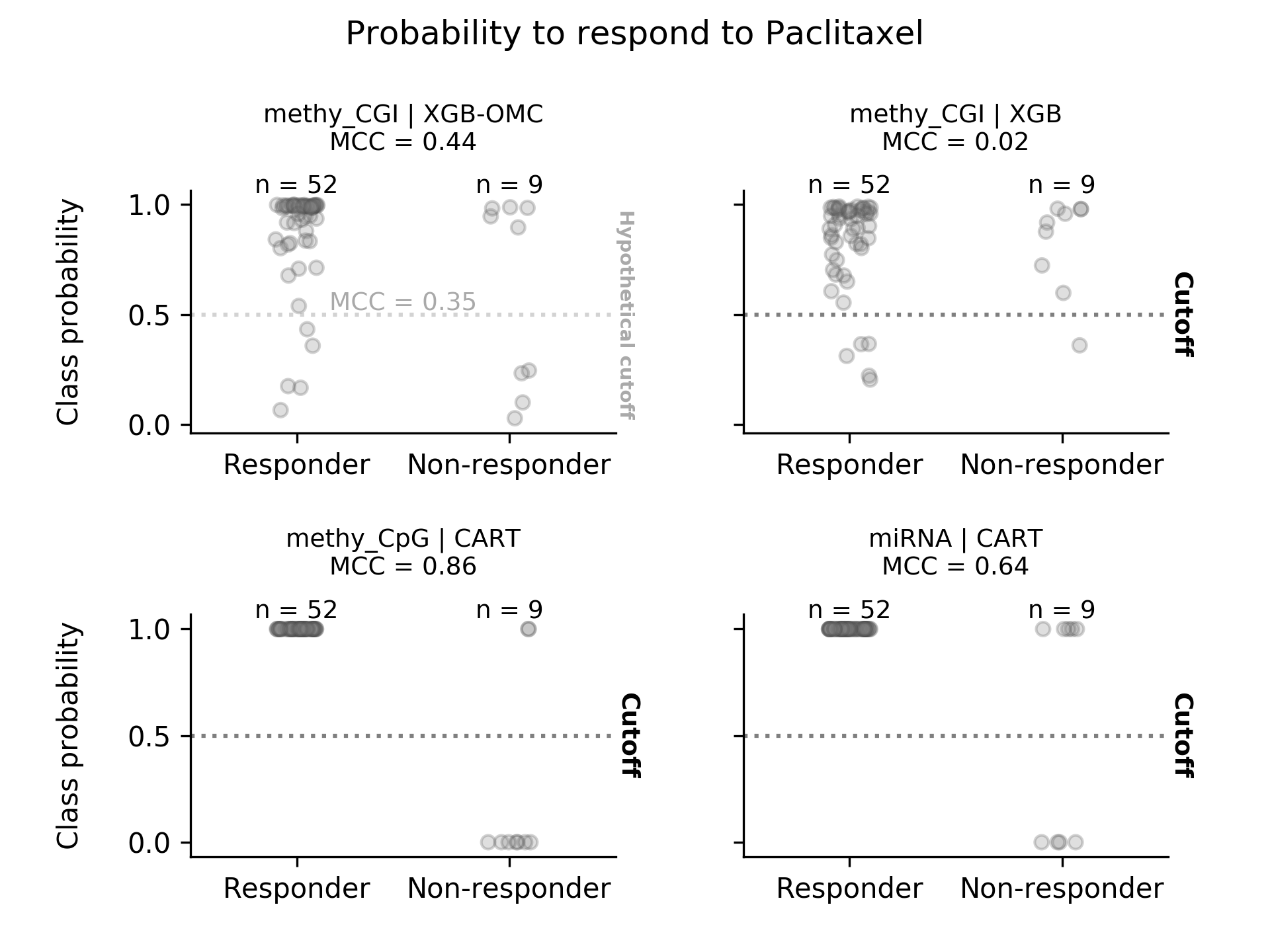
**Supplementary Figure S4**: **The best classifiers allow to see the probability to be paclitaxel-sensitive for each BC patient.** Out of the 60 produced models, we obtained three very predictive classifiers: **(A)** CGI methylation-based XGB-OMC model, **(C)** CpG methylation-based CART model and **(D)** miRNA expression-based CART model. The considered in **(B)** is the all-features version of the model considered in **(A)**. The predictive performance of these models was assessed using several varieties of LOOCV: “standard” for all-features models (CART and XGB) and “nested” for the XGB-OMC. For each type of CV, the predictions obtained from test sets were merged and then MCCs were calculated. This procedure was repeated 10 times setting a different random seed for the employed ML algorithm, then we picked the best case out of the 10 iterations (i.e. the one which reached the highest MCC) and plotted the probabilities to respond to paclitaxel calculated by the corresponding classifier. Each dot of this swarmplot represents a paclitaxel-treated BC patient. In total, there are 61 patients comprising 52 responders and 9 non-responders. Horizontal axes report the original class label assigned to patients, while vertical axes display the calculated probability to respond to paclitaxel. The dashed line shows the cutoff (operating threshold) applied by the classifier to assign the class labels during predictions: for a given instance, if class probability is higher than the cutoff, then the instance is predicted *responder*, otherwise it is predicted *non-responder*. Thus, 1) if a given instance originally labelled *responder* obtained a class probability higher than cutoff (i.e. classified as *responder*), it is counted as a TP; otherwise it is counted as a FN; 2) if a given instance originally labelled non-*responder* obtained a class probability lower than cutoff (i.e. classified as *non-responder*), it is counted as a TN; otherwise it is counted as a FP. As XGB-OMC uses a custom operating threshold for each tested sample (see Supplementary Figure S1), the dashed line corresponds to the default operating threshold that would have been used if no tuning of this hyperparameter to compare the accuracy of predictions.

**(A)** Using customized operating thresholds, the CGI methylation-based XGB-OMC model obtained an MCC of 0.44 (TP = 47, FP = 4, TN = 5, FN = 5). Interestingly, the result is better than using the default cutoff (0.5) which would have given an MCC of 0.35 (TP = 47, FP = 5, TN = 4, FN = 5). Moreover, we can see that anyway this model outperforms **(B)** its all-features version which reached an MCC of 0.02 (TP = 47, FP = 8, TN = 1, FN = 5): this is mainly due to the misclassification of non-responders. **(C)** The CpG methylation- and **(D)** the miRNA expression-based CART models respectively achieved an MCC of 0.86 (TP = 52, FP = 2, TN = 7, FN = 0) and 0.64 (TP = 52, FP = 5, TN = 4, FN = 0). In both cases, all data instances predicted as *responder* got a probability of 1.0 and all data instances predicted as *non-responder* got a probability of 0.0: this is caused by the default parameters applied in *Scikit-learn* for CART algorithm, leading to DTs with pure leaves (see Figure 2).

**
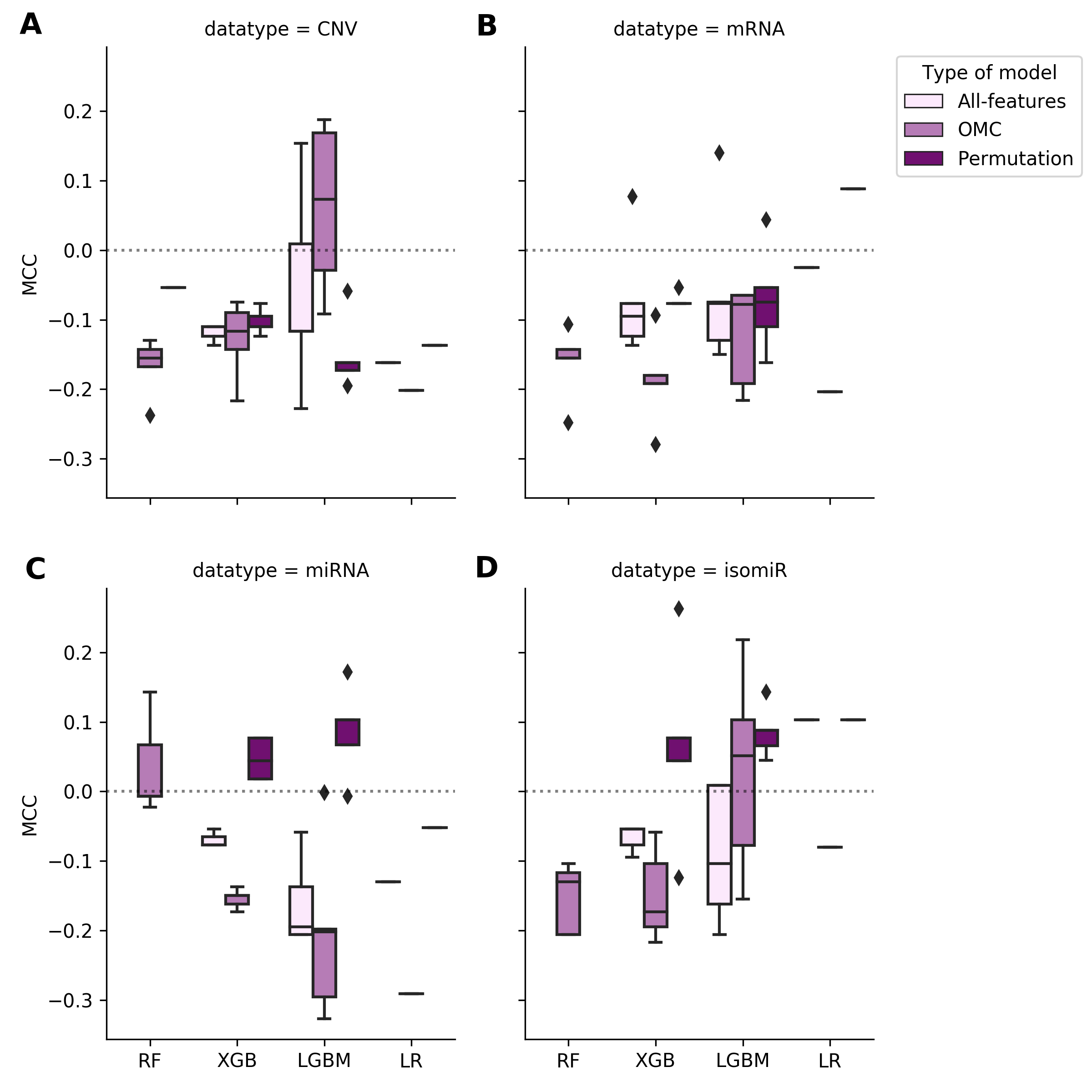
Supplementary**
**Figure S5**: **Predictive performance of** **ML models processing (A) CNV, (B) mRNA, (C) miRNA and (D) isomiR datasets.** Horizontal axes show the employed ML algorithms, while vertical axes display MCC values achieved by classifiers. As five different random seeds were set for ML algorithms, MCCs come from five iterations of cross-validations. Light-pink, medium-pink and indigo boxplots summarise the distributions of MCCs obtained by all-features, OMC and permutations models, respectively. The dashed line shows the expected MCC for random classification. As all-features and permutation models employing RF give undefined MCCs, blanks are found in bins where boxes are supposed to be displayed. ‘CNV’ is short for copy-number variation.


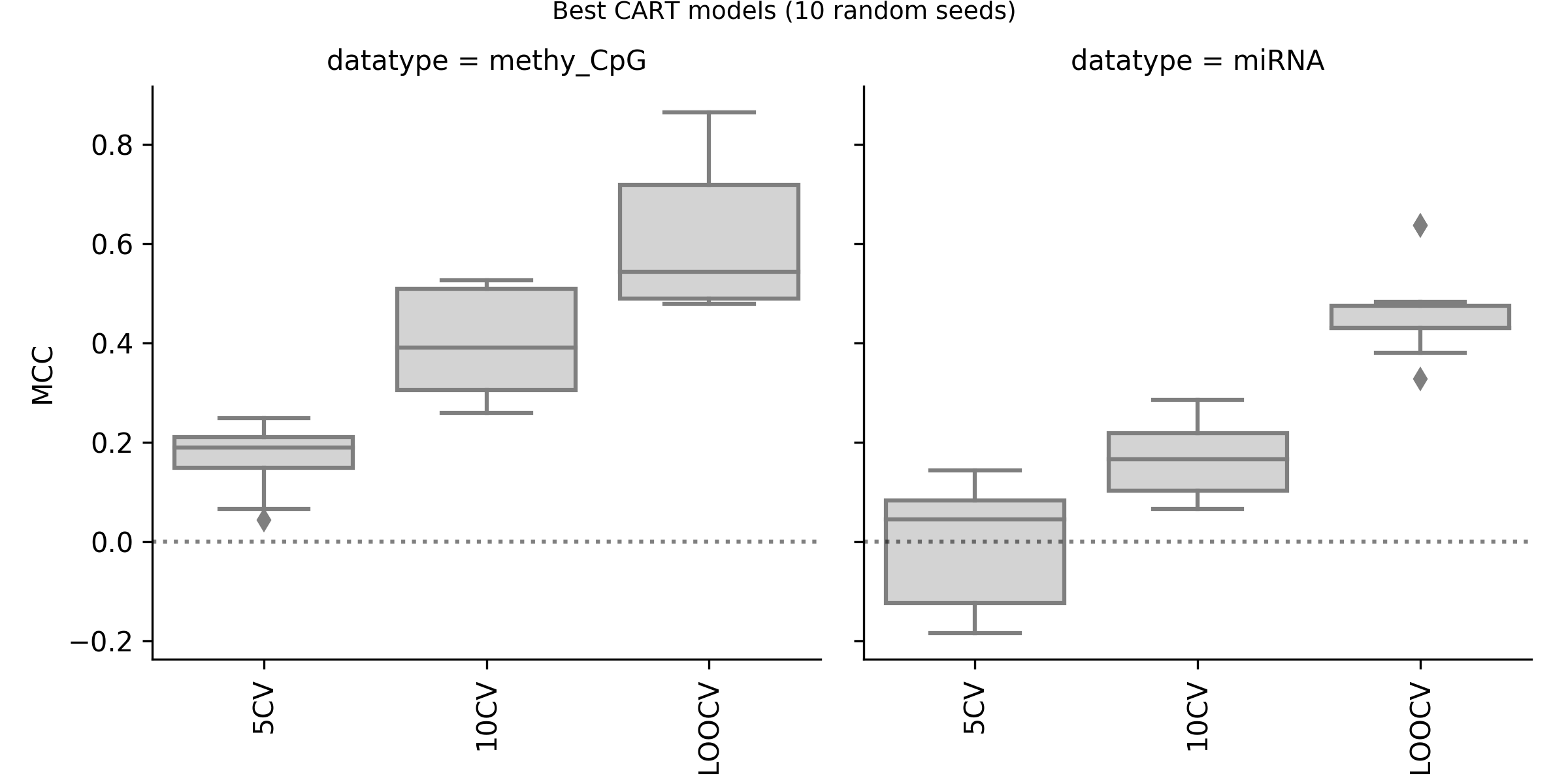


**Supplementary Figure S6**: **Predictive performance of CpG methylation- and miRNA expression-based the best CART models according to the size of the training set.** For the best CART models found from Figure 1C, in addition of LOOCV, we also performed 10-fold cross-validation (10CV), 5-fold cross-validation (5CV) on the same dataset to assess the predictive performance from 10 iterations where different random seeds are set for CART algorithm. For each type of CV, the predictions obtained from test sets were merged and then MCCs were calculated. 5CV, 10CV and LOOCV respectively employ 80%, 90% and 98% of the data in each training fold. At each iteration, the composition of the training folds remains the same. Here, the increasing MCCs along with the increasing sizes of the training sets shows that the predictive performance effectively depends on the availability of the data to train the model. This shows that we can see that these CART models are robust to random seed. ‘methy_CpG’ is short for CpG methylation.

| **ML method - molecular profile** | **Protein coding genes employed by the predictor** | **Linked to cancer and/or drug response** |
| --- | --- | --- |
| **CART - CpG methylation** | MRGPRX4 | The G-protein-coupled receptor MRGPRX4 interacts with the proto-oncogene MAS1 (Jackson et al., 1988; O’Hayre et al., 2013) and have been described as one of the 15 mutational hot spots in colorectal cancer cells (Gylfe et al., 2013). |
|  | SAA2-SAA4 | Serum amyloid A (SAA) proteins are the major acute phase response molecules and considered as a family of apolipoproteins. SAA2-SAA4 is transcribed as a readthrough between the adjunct SAA2 and SAA4 genes (Schulten et al., 2016). SAA expression in BC tissue is associated with poor prognosis (Yang et al., 2016). |
|  | N4BP2L2 | N4BP2L2 stands for ‘NEDD4-binding protein 2-like 2’. NEDD4 plays an important role in development and progression of human cancers (Ye et al., 2014). It has been shown that knock-down in vivo of this protein decreases proliferation, migration, and invasion and improves chemosensitivity to paclitaxel (Song et al., 2018). |
| **XGB-OMC – CGI methylation** | CYP2D6 | Cytochromes P450 participate in deactivation and activation of anticancer drugs (Rodriguez-Antona and Ingelman-Sundberg, 2006; Royer et al., 1996). CYP2D6 is particularly associated with the outcome of tamoxifen-treated BC patients (Abraham et al., 2010, 2011; Damodaran et al., 2012; Hoskins et al., 2009; Sestak et al., 2012; Thompson et al., 2011; Zembutsu et al., 2017). |
|  | MBTPS2 and YY2 | The transcription factor YY2 is encoded by a single exon inserted into the gene locus mbtps2, and a possible shared transcriptional control between YY2 and mbtps2 was suggested (Luo et al., 2006). DNA methylation of yy2 promoter regulates its transcriptional activity which is involved in cell proliferation (Klar et al., 2009; Wu et al., 2017). |
|  | ECRG4/C2orf40 | C2orf40 is a tumour suppressor (Sabatier et al., 2011) frequently silenced in human BC through promoter hypermethylation (Li et al., 2017; Lu et al., 2013). |
|  | IKZF1 | IKZF1 expression allows lung cancer progression (Zhang et al., 2013), whereas it is silenced via hypermethylation in colorectal cancer (Jedi et al., 2018). |
|  | APOBEC4 | APOBEC4 mutagenesis may play a role in cancer development and may be overexpressed in tamoxifen, doxorubicin and etoposide resistant cancer cells (Alexandrov et al., 2013; Burns et al., 2013; Kanu et al., 2016; Kuong and Loeb, 2013; Law et al., 2016; Leonard et al., 2015; Onguru et al., 2016; Seplyarskiy et al., 2016). |
|  | ARPC5 | The regulation of ARPC5 by a tumour-suppressive miRNA contributes to invasion and metastasis in human head and neck squamous cell carcinoma (Seki et al., 2012). |
|  | NCF2 | NCF2 is involved in molecular processes promoting gastric cancer angiogenesis and metastasis (Zhang et al., 2018). In addition, NCF2 has been identified as a target of the tumour suppressor P53 (Italiano et al., 2012). |
|  | SMG7 | SMG7 regulates P53 stability and function in DNA damage stress response (Luo et al., 2016). |
|  | TUBB8 | TUBB8 is one of the tubulin isoforms which have been found to play an important role in paclitaxel resistance in BC (Nami and Wang, 2018; Tame et al., 2017). Moreover, tubulins are directly targeted by paclitaxel (Weaver, 2014). |
| **CART – mature miRNA expression** | miR-942-5p | miR-942-5p is associated to the shorter survival of BC patients, inhibits cell proliferation and metastasis of colorectal cancer and interferes in response to Tumor-necrosis-factor Related Apoptosis Inducing Ligand (TRAIL) treatment (Liu et al., 2014; Shan et al., 2018; Zhang et al., 2017). |
|  | miR-27a-3p | miR-27a-3p takes part in gastric and breast cancer phenotypes and interacts with paclitaxel via a pathway to suppress inflammatory response in sepsis (Mashayekhi et al., 2018; Wu et al., 2018; Yang et al., 2014, 2018). |
|  | miR-217 | miR-217 inhibits proliferation and invasion, predicts prognosis in gastric and pancreatic cancer and is targeted by HOX transcript antisense RNA (HOTAIR) to promote paclitaxel and doxorubicin resistance in gastric cancer cells (Chen et al., 2015; Wang et al., 2018; Yang et al., 2017). |
|  | miR-200c-5p | miR-200c-5p belongs to the miR-200 family dysregulated in invasive breast carcinoma (Aydoǧdu et al., 2012; Tuomarila et al., 2014) and involved in chemosensitivity (Chen and Zhang, 2017). These miRNAs control the expression the tubulin TUBB3 and are associated with paclitaxel response in ovarian cancer (Cittelly et al., 2012; Leskelä et al., 2011). |

**Supplementary Table S15: Connections between features retrieved by the most predictive models and clinical outcomes.** This table summarises the literature review relative to individual factors which constitute molecular signatures retrieved by the most predictive models. The features extracted by CART models are seen in Figure 2, while features extracted by the OMC model are seen in Supplementary Table S14.

**Supplementary tables S1-S11** can be downloaded from http://ballester.marseille.inserm.fr/SupplementaryTables.zip

Python scripts to apply the most predictive models to profiled tumours are available at http://ballester.marseille.inserm.fr/GDC-PaclitaxelBRCA.zip

**REFERENCES**

Abraham, J. E., Maranian, M. J., Driver, K. E., Platte, R., Kalmyrzaev, B., Baynes, C., et al. (2010). CYP2D6 gene variants: Association with breast cancer specific survival in a cohort of breast cancer patients from the United Kingdom treated with adjuvant tamoxifen. *Breast Cancer Res.* 12. doi:10.1186/bcr2629.

Abraham, J. E., Maranian, M. J., Driver, K. E., Platte, R., Kalmyrzaev, B., Baynes, C., et al. (2011). CYP2D6 gene variants and their association with breast cancer susceptibility. *Cancer Epidemiol. Biomarkers Prev.* 20, 1255–1258. doi:10.1158/1055-9965.EPI-11-0321.

Alexandrov, L. B., Nik-Zainal, S., Wedge, D. C., Aparicio, S. a J. R., Behjati, S., Biankin, A. V, et al. (2013). Signatures of mutational processes in human cancer. *Nature* 500, 415–21. doi:10.1038/nature12477.

Aydoǧdu, E., Katchy, A., Tsouko, E., Lin, C. Y., Haldosactue;n, L. A., Helguero, L., et al. (2012). MicroRNA-regulated gene networks during mammary cell differentiation are associated with breast cancer. *Carcinogenesis*. doi:10.1093/carcin/bgs161.

Bengio, Y. (2009). Learning Deep Architectures for AI. *Found. Trends® Mach. Learn.* doi:10.1561/2200000006.

Boughorbel, S., Jarray, F., and El-Anbari, M. (2017). Optimal classifier for imbalanced data using Matthews Correlation Coefficient metric. *PLoS One*. doi:10.1371/journal.pone.0177678.

Breiman, L. (1996). Bagging predictors. *Mach. Learn.* 24, 123–140. doi:10.1007/BF00058655.

Breiman, L. (2001). Random Forests. *Mach. Learn.* 45, 5–32. doi:10.1023/A:1010933404324.

Breiman, L., Friedman, J., Stone, C. J., and Olshen, R. A. (1984). *Classification and Regression Trees*. Chapman and Hall/CRC Available at: http://www.amazon.co.uk/Classification-Regression-Trees-Leo-Breiman/dp/0412048418 [Accessed January 31, 2014].

Burns, M. B., Lackey, L., Carpenter, M. A., Rathore, A., Land, A. M., Leonard, B., et al. (2013). APOBEC3B is an enzymatic source of mutation in breast cancer. *Nature*. doi:10.1038/nature11881.

Cai, Y., Yu, X., Hu, S., and Yu, J. (2009). A Brief Review on the Mechanisms of miRNA Regulation. *Genomics, Proteomics Bioinforma.* doi:10.1016/S1672-0229(08)60044-3.

Cawley, G. C., and Talbot, N. L. C. (2010). On Over-fitting in Model Selection and Subsequent Selection Bias in Performance Evaluation. *J. Mach. Learn. Res.* 11, 2079–2107. Available at: http://www.jmlr.org/papers/v11/cawley10a.html [Accessed July 31, 2017].

Chen, D., Zhang, D., Lu, Y., Chen, L., Zeng, Z., He, M., et al. (2015). microRNA-217 inhibits tumor progression and metastasis by downregulating EZH2 and predicts favorable prognosis in gastric cancer. *Oncotarget* 6. doi:10.18632/oncotarget.3451.

Chen, T., and Guestrin, C. (2016). XGBoost : Reliable Large-scale Tree Boosting System. *arXiv*. doi:10.1145/2939672.2939785.

Chen, Y., and Zhang, L. (2017). Members of the microRNA-200 family are promising therapeutic targets in cancer. *Exp. Ther. Med.* 14, 10–17. doi:10.3892/etm.2017.4488.

Chu, A., Robertson, G., Brooks, D., Mungall, A. J., Birol, I., Coope, R., et al. (2016). Large-scale profiling of microRNAs for the Cancer Genome Atlas. *Nucleic Acids Res.* doi:10.1093/nar/gkv808.

Cittelly, D. M., Dimitrova, I., Howe, E. N., Cochrane, D. R., Jean, A., Spoelstra, N. S., et al. (2012). Restoration of miR-200c to Ovarian Cancer Reduces Tumor Burden and Increases Sensitivity to Paclitaxel. *Mol. Cancer Ther.* doi:10.1158/1535-7163.MCT-12-0463.

Damodaran, S. E., Pradhan, S. C., Umamaheswaran, G., Kadambari, D., Reddy, K. S., and Adithan, C. (2012). Genetic polymorphisms of CYP2D6 increase the risk for recurrence of breast cancer in patients receiving tamoxifen as an adjuvant therapy. *Cancer Chemother. Pharmacol.* 70, 75–81. doi:10.1007/s00280-012-1891-1.

Dang, C. C., Peón, A., and Ballester, P. J. (2018). Unearthing new genomic markers of drug response by improved measurement of discriminative power. *BMC Med. Genomics* 11, 10. doi:10.1186/s12920-018-0336-z.

Fawcett, T. (2006). ScienceDirect.com - Pattern Recognition Letters - An introduction to ROC analysis. *Pattern Recognit. Lett.* doi:10.1016/j.patrec.2005.10.010.

Friedman, J. H. (2001). Greedy function approximation: A gradient boosting machine. *Ann. Stat.* doi:DOI 10.1214/aos/1013203451.

Friedman, J. H. (2002). Stochastic gradient boosting. *Comput. Stat. Data Anal.* doi:10.1016/S0167-9473(01)00065-2.

GDC Reference Files | NCI Genomic Data Commons.

Guo, L., Zheng, L., Zhao, Y., and Wang, Q. (2018). Profiling and Bioinformatic Analyses Indicate Differential circRNA and miRNA/isomiR Expression and Interactions. *Biomed Res. Int.* 2018, 1–9. doi:10.1155/2018/8518563.

Gylfe, A. E., Kondelin, J., Turunen, M., Ristolainen, H., Katainen, R., Pitkänen, E., et al. (2013). Identification of candidate oncogenes in human colorectal cancers with microsatellite instability. *Gastroenterology*. doi:10.1053/j.gastro.2013.05.015.

Han, J., and Moraga, C. (1995). “The influence of the sigmoid function parameters on the speed of backpropagation learning,” in (Springer, Berlin, Heidelberg), 195–201. doi:10.1007/3-540-59497-3_175.

Hanley, J. A., and McNeil, B. J. (1982). The meaning and use of the area under a receiver operating characteristic (ROC) curve. *Radiology*. doi:10.1148/radiology.143.1.7063747.

Hoskins, J. M., Carey, L. A., and McLeod, H. L. (2009). CYP2D6 and tamoxifen: DNA matters in breast cancer. *Nat. Rev. Cancer* 9, 576–586. doi:10.1038/nrc2683.

Italiano, D., Lena, A. M., Melino, G., and Candi, E. (2012). Identification of NCF2/p67phox as a novel p53 target gene. *Cell Cycle* 11, 4589–96. doi:10.4161/cc.22853.

Jackson, T. R., Blair, L. A. C., Marshall, J., Goedert, M., and Hanley, M. R. (1988). The mas oncogene encodes an angiotensin receptor. *Nature*. doi:10.1038/335437a0.

Jedi, M., Young, G. P., Pedersen, S. K., and Symonds, E. L. (2018). Methylation and Gene Expression of BCAT1 and IKZF1 in Colorectal Cancer Tissues. *Clin. Med. Insights Oncol.* 12. doi:10.1177/1179554918775064.

Jianhua, Z. (2018). CNTools: Convert segment data into a region by sample matrix to allow for other high level computational analyses. *Bioconductor Vignette*. doi:10.18129/B9.bioc.CNTools.

Kanu, N., Cerone, M. A., Goh, G., Zalmas, L. P., Bartkova, J., Dietzen, M., et al. (2016). DNA replication stress mediates APOBEC3 family mutagenesis in breast cancer. *Genome Biol.* doi:10.1186/s13059-016-1042-9.

Ke, G., Meng, Q., Wang, T., Chen, W., Ma, W., Liu, T.-Y., et al. (2017). LightGBM: A highly efficient gradient boosting decision tree. *Adv. Neural Inf. Process. Syst.*

Kingma, D. P., and Ba, J. (2014). Adam: A Method for Stochastic Optimization.

Klar, M., Drews, D., and Dame, C. (2009). Transcriptional activity of the novel identified human yy2 promoter is modified by DNA methylation. *Gene* 430, 58–63. doi:10.1016/j.gene.2008.10.013.

Kohavi, R. (1995). A study of cross-validation and bootstrap for accuracy estimation and model selection. *Proc. 14th Int. Jt. Conf. Artif. Intell. - Vol. 2*. doi:10.1067/mod.2000.109031.

Kuong, K. J., and Loeb, L. A. (2013). APOBEC3B mutagenesis in cancer. *Nat. Genet.* 45, 964–965. doi:10.1038/ng.2736.

Law, E. K., Sieuwerts, A. M., Lapara, K., Leonard, B., Starrett, G. J., Molan, A. M., et al. (2016). The DNA cytosine deaminase APOBEC3B promotes tamoxifen resistance in ER-positive breast cancer. *Sci. Adv.* doi:10.1126/sciadv.1601737.

Leonard, B., McCann, J. L., Starrett, G. J., Kosyakovsky, L., Luengas, E. M., Molan, A. M., et al. (2015). The PKC/NF-κB signaling pathway induces APOBEC3B expression in multiple human cancers. *Cancer Res.* doi:10.1158/0008-5472.CAN-15-2171-T.

Leskelä, S., Leandro-García, L. J., Mendiola, M., Barriuso, J., Inglada-Pérez, L., Muñoz, I., et al. (2011). The miR-200 family controls β-tubulin III expression and is associated with paclitaxel-based treatment response and progression-free survival in ovarian cancer patients. *Endocr. Relat. Cancer* 18, 85–95. doi:10.1677/ERC-10-0148.

Li, C., Zhang, P., Jiang, A., Mao, J.-H., and Wei, G. (2017). A short synthetic peptide fragment of human C2ORF40 has therapeutic potential in breast cancer. *Oncotarget* 8, 41963–41974. doi:10.18632/oncotarget.16713.

Liao, Z., Li, D., Wang, X., Li, L., and Zou, Q. (2018). Cancer Diagnosis Through IsomiR Expression with Machine Learning Method. *Curr. Bioinform.* doi:10.2174/1574893611666160609081155.

Liu, F. T., Ting, K. M., and Fan, W. (2005). “Maximizing Tree Diversity by Building Complete-Random Decision Trees,” in (Springer, Berlin, Heidelberg), 605–610. doi:10.1007/11430919_70.

Liu, N., Zuo, C., Wang, X., Chen, T., Yang, D., Wang, J., et al. (2014). miR-942 decreases TRAIL-induced apoptosis through ISG12a downregulation and is regulated by AKT. *Oncotarget* 5. doi:10.18632/oncotarget.2067.

Loshchilov, I., and Hutter, F. (2017). Decoupled Weight Decay Regularization.

Lu, J., Wen, M., Huang, Y., He, X., Wang, Y., Wu, Q., et al. (2013). C2ORF40 suppresses breast cancer cell proliferation and invasion through modulating expression of M phase cell cycle genes. *Epigenetics* 8. doi:10.4161/epi.24626.

Luciano, D. J., Mirsky, H., Vendetti, N. J., and Maas, S. (2004). RNA editing of a miRNA precursor. *RNA*. doi:10.1261/rna.7350304.

Luo, C., Lu, X., Stubbs, L., and Kim, J. (2006). Rapid evolution of a recently retroposed transcription factor YY2 in mammalian genomes. *Genomics* 87, 348–355. doi:10.1016/j.ygeno.2005.11.001.

Luo, H., Cowen, L., Yu, G., Jiang, W., and Tang, Y. (2016). SMG7 is a critical regulator of p53 stability and function in DNA damage stress response. *Cell Discov.* 2. doi:10.1038/celldisc.2015.42.

Mashayekhi, S., Saeidi Saedi, H., Salehi, Z., Soltanipour, S., and Mirzajani, E. (2018). Effects of miR-27a, miR-196a2 and miR-146a polymorphisms on the risk of breast cancer. *Br. J. Biomed. Sci.* 75, 76–81. doi:10.1080/09674845.2017.1399572.

Matthews, B. W. (1975). Comparison of the predicted and observed secondary structure of T4 phage lysozyme. *Biochim. Biophys. Acta - Protein Struct.* 405, 442–451. doi:10.1016/0005-2795(75)90109-9.

Murphy, K. P. (2012). *Machine learning: a probabilistic perspective (adaptive computation and machine learning series)*.

Nami, B., and Wang, Z. (2018). Genetics and expression profile of the Tubulin gene superfamily in breast cancer subtypes and its relation to taxane resistance. *Cancers (Basel).* doi:10.1016/j.ijpharm.2014.10.053.

Nguyen, L., Naulaerts, S., Bomane, A., Bruna, A., Ghislat, G., and Ballester, P. (2018). Machine learning models to predict in vivo drug response via optimal dimensionality reduction of tumour molecular profiles. *bioRxiv*, 277772. doi:10.1101/277772.

O’Hayre, M., Vázquez-Prado, J., Kufareva, I., Stawiski, E. W., Handel, T. M., Seshagiri, S., et al. (2013). The emerging mutational landscape of G proteins and G-protein-coupled receptors in cancer. *Nat. Rev. Cancer*. doi:10.1038/nrc3521.

Olshen, A. B., Venkatraman, E. S., Lucito, R., and Wigler, M. (2004). Circular binary segmentation for the analysis of array-based DNA copy number data. *Biostatistics* 5, 557–572. doi:10.1093/biostatistics/kxh008.

Onguru, O., Yalcin, S., Rosemblit, C., Zhang, P. J., Kilic, S., and Gunduz, U. (2016). APOBEC3B expression in drug resistant MCF-7 breast cancer cell lines. *Biomed. Pharmacother.* doi:10.1016/j.biopha.2016.02.004.

Pedregosa, F., Varoquaux, G., Gramfort, A., Michel, V., Thirion, B., Grisel, O., et al. (2011). Scikit-learn: Machine Learning in Python. *J. Mach. Learn. Res.* 12, 2825–2830.

Ranstam, J., Cook, J. A., and Collins, G. S. (2016). Clinical prediction models. *Br. J. Surg.* doi:10.1002/bjs.10242.

Van Rijsbergen, C. J. (1979). *Information Retrieval, 2nd edition*. doi:10.1016/j.pestbp.2006.07.008.

Rodriguez-Antona, C., and Ingelman-Sundberg, M. (2006). Cytochrome P450 pharmacogenetics and cancer. *Oncogene* 25, 1679–1691. doi:10.1038/sj.onc.1209377.

Royer, I., Monsarrat, B., Sonnier, M., Wright, M., and Cresteil, T. (1996). Metabolism of docetaxel by human cytochromes P450: Interactions with paclitaxel and other antineoplastic drugs. *Cancer Res.* 56, 58–65.

Sabatier, R., Finetti, P., Adelaide, J., Guille, A., Borg, J. P., Chaffanet, M., et al. (2011). Down-regulation of ECRG4, a candidate tumor suppressor gene, in human breast cancer. *PLoS One*. doi:10.1371/journal.pone.0027656.

Saeys, Y., Inza, I., and Larrañaga, P. (2007). A review of feature selection techniques in Bioinformatics. *Bioinformatics* 23, 2507–2517. doi:10.1093/bioinformatics/btm344.

Schulten, H. J., Hussein, D., Al-Adwani, F., Karim, S., Al-Maghrabi, J., Al-Sharif, M., et al. (2016). Microarray expression profiling identifies genes, including cytokines, and biofunctions, as diapedesis, associated with a brain metastasis from a papillary thyroid carcinoma. *Am. J. Cancer Res.*

Seki, N., Nohata, N., Watanabe-Takano, H., Yoshino, H., Hidaka, H., Fujimura, L., et al. (2012). Actin-related protein 2/3 complex subunit 5 (ARPC5) contributes to cell migration and invasion and is directly regulated by tumor-suppressive microRNA-133a in head and neck squamous cell carcinoma. *Int. J. Oncol.* 40, 1770–1778. doi:10.3892/ijo.2012.1390.

Seplyarskiy, V. B., Soldatov, R. A., Popadin, K. Y., Antonarakis, S. E., Bazykin, G. A., and Nikolaev, S. I. (2016). APOBEC-induced mutations in human cancers are strongly enriched on the lagging DNA strand during replication. *Genome Res.* 26, 174–182. doi:10.1101/gr.197046.115.

Seshan, V. E., and Olshen, A. B. (2014). DNAcopy : A Package for Analyzing DNA Copy Data. *Bioconductor Vignette*.

Sestak, I., Kealy, R., Nikoloff, M., Fontecha, M., Forbes, J. F., Howell, A., et al. (2012). Relationships between CYP2D6 phenotype, breast cancer and hot flushes in women at high risk of breast cancer receiving prophylactic tamoxifen: Results from the IBIS-I trial. *Br. J. Cancer* 107, 230–233. doi:10.1038/bjc.2012.278.

Shan, Z., An, N., Qin, J., Yang, J., Sun, H., and Yang, W. (2018). Long non-coding RNA Linc00675 suppresses cell proliferation and metastasis in colorectal cancer via acting on miR-942 and Wnt/β-catenin signaling. *Biomed. Pharmacother.* 101, 769–776. doi:10.1016/j.biopha.2018.02.123.

Song, Y. H., Zhang, C. Q., Chen, F. F., and Lin, X. Y. (2018). Upregulation of Neural Precursor Cell Expressed Developmentally Downregulated 4-1 is Associated with Poor Prognosis and Chemoresistance in Lung Adenocarcinoma. *Chin. Med. J. (Engl).* doi:10.4103/0366-6999.221262.

Srivastava, N., Hinton, G., Krizhevsky, A., Sutskever, I., and Salakhutdinov, R. (2014). Dropout: A simple way to prevent neural networks from overfitting. *J. Mach. Learn. Res.*

Svetnik, V., Liaw, A., Tong, C., Culberson, J. C., Sheridan, R. P., and Feuston, B. P. (2003). Random forest: a classification and regression tool for compound classification and QSAR modeling. *J. Chem. Inf. Comput. Sci.* 43, 1947–58. doi:10.1021/ci034160g.

Tame, M. A., Manjón, A. G., Belokhvostova, D., Raaijmakers, J. A., and Medema, R. H. (2017). TUBB3 overexpression has a negligible effect on the sensitivity to taxol in cultured cell lines. *Oncotarget*. doi:10.18632/oncotarget.17740.

Telonis, A. G., Loher, P., Jing, Y., Londin, E., and Rigoutsos, I. (2015). Beyond the one-locus-one-miRNA paradigm: microRNA isoforms enable deeper insights into breast cancer heterogeneity. *Nucleic Acids Res.* doi:10.1093/nar/gkv922.

Thompson, A. M., Johnson, A., Quinlan, P., Hillman, G., Fontecha, M., Bray, S. E., et al. (2011). Comprehensive CYP2D6 genotype and adherence affect outcome in breast cancer patients treated with tamoxifen monotherapy. *Breast Cancer Res. Treat.* 125, 279–287. doi:10.1007/s10549-010-1139-x.

Tuomarila, M., Luostari, K., Soini, Y., Kataja, V., Kosma, V. M., and Mannermaa, A. (2014). Overexpression of microRNA-200c predicts poor outcome in patients with PR-negative breast cancer. *PLoS One* 9. doi:10.1371/journal.pone.0109508.

Varma, S., and Simon, R. (2006). Bias in error estimation when using cross-validation for model selection. *BMC Bioinformatics* 7, 91. doi:10.1186/1471-2105-7-91.

Wang, H., Qin, R., Guan, A., Yao, Y., Huang, Y., Jia, H., et al. (2018). HOTAIR enhanced paclitaxel and doxorubicin resistance in gastric cancer cells partly through inhibiting miR-217 expression. *J. Cell. Biochem.* 119, 7226–7234. doi:10.1002/jcb.26901.

Weaver, B. A. (2014). How Taxol/paclitaxel kills cancer cells. *Mol. Biol. Cell* 25, 2677–81. doi:10.1091/mbc.E14-04-0916.

Wu, J., Sun, Z., Sun, H., and Li, Y. (2018). MicroRNA-27a promotes tumorigenesis via targeting AKT in triple negative breast cancer. *Mol. Med. Rep.* 17, 562–570. doi:10.3892/mmr.2017.7886.

Wu, X. N., Shi, T. T., He, Y. H., Wang, F. F., Sang, R., Ding, J. C., et al. (2017). Methylation of transcription factor YY2 regulates its transcriptional activity and cell proliferation. *Cell Discov.* 3. doi:10.1038/celldisc.2017.35.

Xu, X., Gu, H., Wang, Y., Wang, J., and Qin, P. (2019). Autoencoder Based Feature Selection Method for Classification of Anticancer Drug Response. *Front. Genet.* 10, 233. doi:10.3389/fgene.2019.00233.

Yang, J., Zhang, H. F., and Qin, C. F. (2017). MicroRNA-217 functions as a prognosis predictor and inhibits pancreatic cancer cell proliferation and invasion via targeting E2F3. *Eur. Rev. Med. Pharmacol. Sci.* 21, 4050–4057.

Yang, M., Liu, F., Higuchi, K., Sawashita, J., Fu, X., Zhang, L., et al. (2016). Serum amyloid A expression in the breast cancer tissue is associated with poor prognosis. *Oncotarget*. doi:10.18632/oncotarget.8561.

Yang, Q., Jie, Z., Ye, S., Li, Z., Han, Z., Wu, J., et al. (2014). Genetic variations in miR-27a gene decrease mature miR-27a level and reduce gastric cancer susceptibility. *Oncogene* 33, 193–202. doi:10.1038/onc.2012.569.

Yang, Q., Zhang, D., Li, Y., Li, Y., and Li, Y. (2018). Paclitaxel alleviated liver injury of septic mice by alleviating inflammatory response via microRNA-27a/TAB3/NF-κB signaling pathway. *Biomed. Pharmacother.* 97, 1424–1433. doi:10.1016/j.biopha.2017.11.003.

Ye, X., Wang, L., Shang, B., Wang, Z., and Wei, W. (2014). NEDD4: a promising target for cancer therapy. *Curr. Cancer Drug Targets* 14, 549–56.

Zembutsu, H., Nakamura, S., Akashi-Tanaka, S., Kuwayama, T., Watanabe, C., Takamaru, T., et al. (2017). Significant effect of polymorphisms in CYP2D6 on response to tamoxifen therapy for breast cancer: A prospective multicenter study. *Clin. Cancer Res.* 23, 2019–2026. doi:10.1158/1078-0432.CCR-16-1779.

Zhang, J. X., Chen, Z. H., Chen, D. L., Tian, X. P., Wang, C. Y., Zhou, Z. W., et al. (2018). LINC01410-miR-532-NCF2-NF-kB feedback loop promotes gastric cancer angiogenesis and metastasis. *Oncogene* 37, 2660–2675. doi:10.1038/s41388-018-0162-y.

Zhang, K., Wang, Y. W., Wang, Y. Y., Song, Y., Zhu, J., Si, P. C., et al. (2017). Identification of microRNA biomarkers in the blood of breast cancer patients based on microRNA profiling. *Gene* 619, 10–20. doi:10.1016/j.gene.2017.03.038.

Zhang, Z., Xu, Z., Wang, X., Wang, H., Yao, Z., Mu, Y., et al. (2013). Ectopic ikaros expression positively correlates with lung cancer progression. *Anat. Rec.* 296, 907–913. doi:10.1002/ar.22700.

Zhou, Y., Cahya, S., Combs, S. A., Nicolaou, C. A., Wang, J., Desai, P. V., et al. (2019). Exploring Tunable Hyperparameters for Deep Neural Networks with Industrial ADME Data Sets. *J. Chem. Inf. Model.* 59, 1005–1016. doi:10.1021/acs.jcim.8b00671.
